# Recent Progress and Prospect of Metal–Organic Framework-Based Nanozymes in Biomedical Application

**DOI:** 10.3390/nano14030244

**Published:** 2024-01-23

**Authors:** Anupriya Baranwal, Shakil Ahmed Polash, Vijay Kumar Aralappanavar, Bijay Kumar Behera, Vipul Bansal, Ravi Shukla

**Affiliations:** 1 Sir Ian Potter NanoBioSensing Facility, NanoBiotechnology Research Laboratory, School of Science, RMIT University, Melbourne, VIC 3000, Australiavipul.bansal@rmit.edu.au (V.B.); 2 NanoBiosensor Laboratory, Aquatic Environmental Biotechnology and Nanotechnology Division, ICAR-Central Inland Fisheries Research Institute, Barrackpore, Kolkata 700120, West Bengal, India; 3 Centre for Advanced Materials & Industrial Chemistry, RMIT University, Melbourne, VIC 3000, Australia

**Keywords:** metal–organic framework, MOF, nanozyme, enzyme-mimic, biosensor, therapeutics, cancer therapy, antimicrobial, anti-inflammatory

## Abstract

A nanozyme is a nanoscale material having enzyme-like properties. It exhibits several superior properties, including low preparation cost, robust catalytic activity, and long-term storage at ambient temperatures. Moreover, high stability enables repetitive use in multiple catalytic reactions. Hence, it is considered a potential replacement for natural enzymes. Enormous research interest in nanozymes in the past two decades has made it imperative to look for better enzyme-mimicking materials for biomedical applications. Given this, research on metal–organic frameworks (MOFs) as a potential nanozyme material has gained momentum. MOFs are advanced hybrid materials made of inorganic metal ions and organic ligands. Their distinct composition, adaptable pore size, structural diversity, and ease in the tunability of physicochemical properties enable MOFs to mimic enzyme-like activities and act as promising nanozyme candidates. This review aims to discuss recent advances in the development of MOF-based nanozymes (MOF-NZs) and highlight their applications in the field of biomedicine. Firstly, different enzyme-mimetic activities exhibited by MOFs are discussed, and insights are given into various strategies to achieve them. Modification and functionalization strategies are deliberated to obtain MOF-NZs with enhanced catalytic activity. Subsequently, applications of MOF-NZs in the biosensing and therapeutics domain are discussed. Finally, the review is concluded by giving insights into the challenges encountered with MOF-NZs and possible directions to overcome them in the future. With this review, we aim to encourage consolidated efforts across enzyme engineering, nanotechnology, materials science, and biomedicine disciplines to inspire exciting innovations in this emerging yet promising field.

## 1. Introduction

Enzymes are naturally occurring biological catalysts that accelerate the rate of biochemical reactions without being consumed in the process. They are almost always proteins and only sometimes RNA (ribozymes). As strong biological catalysts, natural enzymes exhibit high catalytic activity and substrate specificity, enabling them to be applied in fields including, but not limited to, bioengineering, food processing, biomedicine, chemical production, and environmental remediation [1,2,3]. Despite these promising merits, natural enzymes suffer from intrinsic limitations of cumbersome preparation, complex and expensive purification, low recyclability, difficult tunability, and poor stability. Given this, extensive efforts have been made to develop artificial enzymes that can overcome these shortcomings and replace natural enzymes [4,5,6]. Ever since the discovery of magnetic nanoparticles with intrinsic peroxidase (POX)-like activity [7], various nanomaterials such as metal and metal oxide nanoparticles, metal–organic frameworks (MOFs), carbon nanomaterials, and their composites have been investigated for enzyme-like activities [8,9,10,11,12,13,14,15]. These enzymes mimicking catalytic nanomaterials are being referred to as ‘nanozymes’ by following a similar nomenclature pattern as that of DNAzymes, ribozymes, synzymes, etc. Unlike natural enzymes and other conventional artificial enzymes, nanozymes can be easily synthesized and mass-produced at a lower cost.

Moreover, the unique physicochemical properties of nanozymes endow them with an ease of tunability, high catalytic activity and stability, and longer shelf-life [11,16]. These merits and advancements in nano-biotechnology have pushed nanozymes to the forefront of developing new biosensors, theranostics, bioimaging techniques, antimicrobials, and environmental treatments [17,18,19,20,21]. Despite achieving such commendable progress in a relatively short span, only certain nanozymes can perform efficiently in vitro catalysis. In contrast, others show inadequate performance and poor catalytic reaction selectivity due to the influencing intrinsic (pH, temperature, redox condition, and oxygen level) or extrinsic (light, magnetic field, heat, and ultrasound) parameters [22,23,24]. In addition, several other factors, such as the size, morphology, elemental composition, surface modification, and crystal structure of nanozymes, could also influence their catalytic performance [22]. Therefore, it is imperative to develop nanozymes with precise catalytic mechanisms and active sites to meet the standards of natural enzymes and broaden their scope of biomedical applications [25,26].

**Figure 1:**
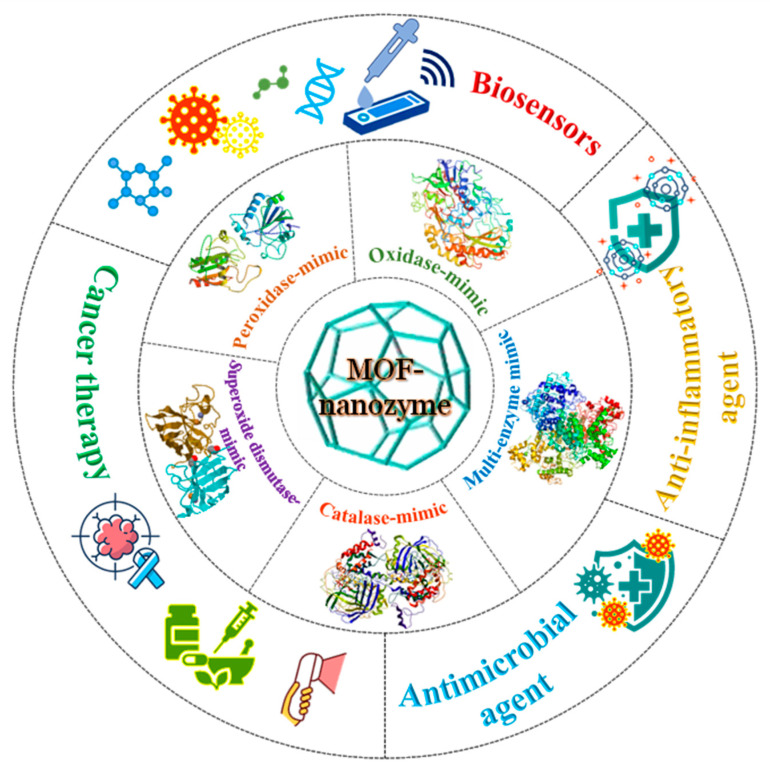
Schematic illustration summarizing types of MOF-NZs and their potential applications in various biomedical fields.

MOFs are an emerging class of porous, crystalline, inorganic–organic hybrid materials that consist of coordinated metal ions/clusters (metal nodes) and organic ligands [27,28,29]. Owing to the strong coordination between metal nodes and bridging ligands, MOFs with unique structures and flexible properties have emerged. These include the presence of (i) molecular/atomic level catalytic sites, (ii) multiple channels and ultrahigh porosity, (iii) abundant catalytic active sites, (iv) synthesis and chemical tunability, and (v) high stability [30,31,32,33]. On account of these properties, MOFs have been applied in fields ranging from chemical catalysis, biochemical analysis, sensing, energy storage, gas separation, antimicrobials, drug and gene delivery, and so on [34,35,36,37,38,39]. In recent years, MOFs have become promising candidates in bio-catalysis due to their intrinsic nanozyme activity and ability to facilitate enzyme immobilization [34,40]. Compared to traditional nanozymes, MOF-NZs possess the potential to create a diverse range of nanozymes due to the versatility of metal ions and organic linkers. Their regularly arranged unit cells offer active catalytic sites with improved enzyme mimicry, and their porous structure aids in directing substrates to these active sites [41]. Moreover, the modular nature of MOFs promotes the incorporation of various metal ions or clusters, allowing for precise control over catalytic properties [42]. By combining the therapeutic and catalytic functionalities in a single material, MOFs can also serve as excellent drug delivery carriers and play a crucial role in theranostics [43]. Owing to this, different types of MOFs, including unmodified/pristine MOFs, modified MOFs, derived MOFs, and MOFs conjugated with natural enzymes, have been investigated for their nanozyme activity [24,44,45]. Reportedly, pristine MOFs, such as Material Institute of Lavoisier (MIL)-53, MIL-88, MIL-100, and MIL-10, can exhibit excellent catalytic activity by either mimicking the natural enzymes’ active sites or by polyvalent elements [46]. Their modified forms have been developed to add chemical functionality to the MOF interior or onto its surface by using metal [47,48] and metal oxide [47,48] nanoparticles. To further improve the catalytic activity of MOFs, their derivatives have been synthesized by employing thermolysis [49], etching [50], or pyrolysis methods [51]. In addition to this, MOF-encapsulated natural enzymes have been prepared to increase their stability under harsh conditions and exhibit superior catalytic activity than that of pristine MOFs [52,53]. Thanks to the diversity and structural tunability of MOF-based materials, MOF-based nanozymes (MOF-NZs) simulating a variety of enzyme-like activities have been developed and applied in various biomedical fields (Figure 1).

While several reviews on the nanozyme activity of MOFs exist in the literature, most of these reviews have primarily focused on general classifications, construction mechanisms, and applications of MOF-NZs within specific domains, such as biosensing [34,54,55], cancer therapy [56,57], or theranostics [43]. MOF-NZs, however, can be utilized in a rather large number of applications and a review encompassing these applications would imply their tremendous potential and be timely. In view of this, we have reviewed the advancements in MOF-NZ development with a key focus on their application in different biosensing (colorimetric, fluorescence, and electrochemical) and therapeutic (chemotherapy, photothermal, photodynamic, anti-inflammatory, and antimicrobial therapy) domains. Modification and functionalization strategies are discussed to enhance their catalytic activities and thereby improve their applied performance. Two extensive tables highlighting different aspects of MOF-NZs in biosensing and therapeutic applications have also been deliberated. Finally, the challenges in the field are highlighted to reveal existing research gaps and possible directions are provided to overcome them in the future.

## 2. Types of MOF-NZs

MOF-NZs exhibit high catalytic activity compared to that of pristine nanozymes due to their structural components: metal nodes (e.g., Fe, Cu, Ce, Mn, etc.) and organic ligands (e.g., 2-methyl imidazole, 2,2′-dithiosalicylic acid, etc.) [58], where the former acts as a redox couple (e.g., Fe^3+^/Fe^2+^, Cu^2+^/Cu, Ce^4+^/Ce^3+^, Mn^2+^/Mn) and the latter acts as a redox mediator for donating and accepting electrons from one substrate to another [59]. Over the last few years, numerous MOFs have been successfully developed to mimic the peroxidase (POX), oxidase (OX), superoxide dismutase (SOD), and catalase (CAT) activities of natural enzymes. Interestingly, some of the developed MOFs could also mimic two or more enzymatic activities under the same conditions or in a different environment. The following section is compiled to summarize different types of MOF-NZs reported in recent years.

### 2.1. Peroxidase-Mimic

POX is an oxidoreductase enzyme that catalyzes the oxidation of substrates by employing peroxide as an electron acceptor. POX activity is mainly attributed to Fenton-reactions, wherein the chromogenic substrates, such as 3,3′,5,5′-tetramethylbenzidine (TMB), 2,2-azinobis(3-ethylbenzothiazoline)-6-sulfonic acid (ABTS), and o-phenylenediamine (OPD) (electron donors), are oxidized in the presence of H_2_O_2_ (electron acceptor). A natural POX such as horse-radish peroxidase (HRP) has a coordinated heme molecule that serves as a catalytic active site. To mimic the structural and functional attributes of HRP, MOFs have been developed with a hybrid array of central metal nodes acting as active catalytic sites, and organic linkers as structural ligands [6]. For example, in iron-porphyrin MOFs, the iron-porphyrin structure is employed as a structural motif to mimic the heme-like active center and function as a POX [60].

#### 2.1.1. Pristine MOF Nanozymes

**Figure 2:**
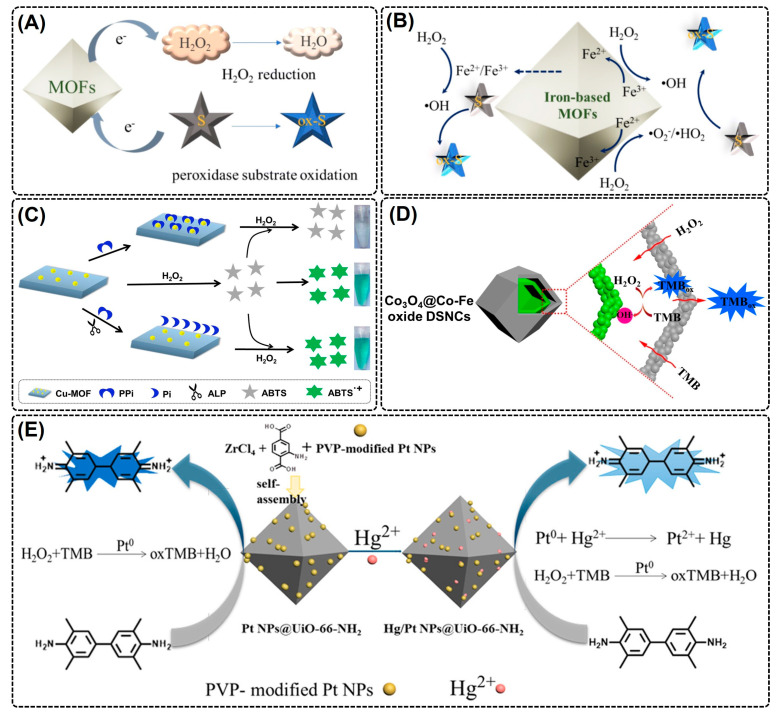
POX-mimic activity of MOF. Schematic illustration of (**A**) generic and (**B**) Fe-based pristine MOFs exhibiting POX-mimic activity for chromogenic TMB substrate (S) oxidation in the presence of H_2_O_2_. Reproduced with permission from [55]; (**C**) Cu(II)-MOF, wherein the active metal nodes (Cu(II)) acted as POX-mimic and oxidized the chromogenic substrate ABTS in the presence of H_2_O_2_. Reproduced with permission from [62]; (**D**) POX-mimic activity of Co_3_O_4_@CO-Fe oxide DNSC. Reproduced with permission from [65]; (**E**) POX-mimic activity of Pt NPs functionalized UiO-66-NH_2_ MOF. Reproduced with permission from [66].

To date, numerous MOFs containing active redox couples as metal nodes have been developed to mimic the POX activity. To date, several MOFs have been reported to show intrinsic peroxidase-mimic activity, such as Material Institute of Lavoisier (MIL)-53 (20), MIL-100(Fe) (21), MIL-100(Al)-NH_2_ (19), and Hong Kong University of Science and Technology (HKUST)-1 [61]. These redox couples catalytically activate H_2_O_2_ by employing a Fenton-like reaction to produce a hydroxyl radical (^•^OH) and thus render MOFs to oxidize chromogenic POX substrates (Figure 2A,B) [55,62]. In one such report, Cu(II)-MOFs were designed using 4,4′-bipyridine as organic linkers and Cu(II) as the active metallic center by employing a self-assembly strategy [62]. The Cu(II) metal nodes acted as a POX-mimic and oxidized the chromogenic substrate ABTS in the presence of H_2_O_2_ (Figure 2C). Moreover, the positively charged Cu(II)-MOF showed a higher electrostatic affinity towards electronegative ABTS (lower K_m_) than that of HRP. Similarly, MIL-68 and MIL-100 were developed with Fe(III) as an active metal catalyst using a solvothermal process, wherein both MOFs mimicked POX by oxidizing the chromogenic substrate TMB to produce a deep blue color product in the presence of H_2_O_2_ [63]. While these pristine MOFs have certain advantages over natural enzymes, they possess limited catalytic active sites, exhibit poor affinity and specificity to substrates, and display poor catalytic performance. This warrants a further improvement in the structural attributes of MOFs during synthesis or post-synthesis modification.

#### 2.1.2. Modified MOF Nanozymes

Metal nodes or organic linkers of pristine MOFs are modified either during or post-synthesis to develop highly performing MOF-derived nanozymes [6]. There are various means to achieve MOF derivatives: (i) metal ions substitution/exchange, (ii) heteroatom doping, (iii) organic ligand substitution, (iv) the introduction of new functional groups, (v) using MOF as a sacrificial template to develop nanomaterials with enzyme-mimic activity, and (vi) the introduction of nanoparticles. Interestingly, the combination of one or more strategies has been shown to increase the catalytic performance of MOFs through (i) structure and size modification, (ii) the addition of new recognition sites, and (iii) an increase in the number of active sites. Using the heteroatom doping strategy, Cheng et al. (2022) doped non-catalytic Ni(II) nodes into 1-D metal oxide octahedral chains of MOF-53(Fe) by using two facile procedures: solvothermal synthesis and hydrogen reduction [64]. As a result, the designed bimetallic Ni_x_-Fe-MOF posed superior POX-mimic activity, increased the number of coordination unsaturated sites, and enhanced substrate affinity. Moreover, Fe(III)/Fe(II) in bimetallic Ni_x_-Fe-MOF is involved in the reversible catalysis of H_2_O_2_. Another approach to obtaining MOF-derived nanozymes is by using MOFs as sacrificial templates under various annealing conditions. Using (ZIF)-67 as a substrate template, Chen et al. (2020) developed a Co_3_O_4_@CO-Fe oxide double-shelled hybrid nanocage (DSNC) by combining an anion exchange reaction between the zeolitic imidazolate framework (ZIF)-67 and [Fe(CN)_6_]^3−^ followed by low-temperature pyrolysis at 350 °C for 2 h [65]. On one hand, Co_3_O_4_ and Fe oxide rendered a derived MOF with excellent POX-like activity, and on the other hand, the Co_3_O_4_@CO-Fe oxide porous structure provided a confined nano-framework for substrate-catalyst reactions and additional active sites for substrate catalysis (Figure 2D). Owing to the excellent catalytic potential of the developed MOF composite, researchers could detect H_2_O_2_ with high sensitivity and selectivity in the serum samples. Thus, from the aforementioned studies, it is evident that the MOF modification by heteroatom doping would be a feasible strategy to increase the overall catalytic performance and substrate specificity of the pristine MOF.

The enzyme-mimic properties of various metal/metal oxide NPs (e.g., Au, Ag, iron oxide, zinc oxide, etc.) have been explored for their wide range of applications in nanozyme-based biomedicine. However, limited structural attributes, a low catalytic performance due to inefficiently exposed active sites, and poor stability (homo-or/and hetero-aggregation) often limit their wide range of practical applications in complex natural systems [6]. To address this, MOFs with large porous structures are explored as a structural scaffold for incorporating POX-mimic metal/metal oxide NPs, which exhibited a synergistic POX-mimic catalytic activity, excellent substrate affinity, and high NP stability. Li et al. (2017) functionalized the POX-mimic activity of Pt NPs with a catalytically inert UiO-66-NH_2_ MOF for the detection and removal of Hg(II) from the water samples [66]. The dispersed NPs exhibit higher POX-like activity relative to pristine Pt NPs. Moreover, the porous microstructure of the MOF increased the substrate-catalyst interaction with better catalytic activity and stability of the Pt NPs of the developed MOF (Figure 2E). Similarly, the dispersion of Au NPs on the NH_2_-MIL-125(Ti) MOF formed a multifunctional AuNPs@NH_2_-MIL-125(Ti) with a higher substrate affinity and synergistic POX-mimic activity. The AuNPs@NH_2_-MIL-125(Ti) demonstrated a highly sensitive and selective detection of H_2_O_2_, cysteine, and Hg(II) ions from the water system [67]. Dang et al. (2020) developed a bimetallic (Mn, Fe) MOF and functionalized it with gold nanoparticles (AuNPs) and anchored carbon-nanotubes (CNTs) (Au/MOF(Fe, Mn)/CNTs) as an excellent POX-mimic [68]. The developed composite nanozyme was then employed to mediate the sensitive detection of glucose, H_2_O_2_, and sulfadimethoxine. The presence of Mn and Fe in an MOF composite not only increased the number of catalytic active sites but also enhanced electron transfer between MOF, Au, and CNTs, thereby resulting in a two-to-eight-times higher POX-mimic activity than that of a pristine MOF. On further analyses, the bimetallic MOF composites displayed a higher substrate affinity (K_m_ = 0.33 mM) and reaction velocity (V_max_ = 17.65 × 10^−7^ M s^−1^) for H_2_O_2_ than that of natural HRP (K_m_ = 3.7 mM, V_max_ = 0.87 × 10^−7^ M s^−1^), suggesting that a developed MOF composite could successfully serve as an HRP surrogate in biosensor platforms. Thus, the functionalization of MOFs with metal/metal oxide nanozymes resulted in synergistic POX-mimic activity with significant catalytic performance, superior surface characteristics, enhanced substrate availability and affinity, and reusability.

### 2.2. Oxidase-Mimic

OX-mimic nanozymes directly activate dissolved O_2_ to form ^•^O_2_ radicals, preventing the use of unstable and destructive H_2_O_2_ as an oxidant [46]. Owing to their excellent O_2_ reduction activity, the noble metal NPs and their composites have displayed OX-mimic catalytic activity [69,70]. However, their high synthesis cost and limited availability hindered their widespread application. Some of the transition metal oxide NPs, such as CeO_2_ NPs, MnO_2_ NPs, and NiO_2_ NPs, have also shown similar OX-mimic activity [71,72,73,74], but their catalytic activity is relatively poor compared to natural enzymes [75]. Therefore, the need for cost-effective OX-mimic nanozymes with high catalytic activity and substrate specificity is of great interest. Recently, MOFs have gained considerable attention as OX-mimic nanozymes due to their small porous structure and capability of eliminating interference, thereby endowing them with high catalytic activity and substrate specificity.

#### 2.2.1. Pristine MOF Nanozymes

Pristine MOFs, such as Ce-MOFs and Cu-MOFs, have been exploited as OX-mimics for MOF-based sensor development and therapeutic applications [76,77,78]. In one such report, Xiong et al. (2015) developed an OX-mimic MOF by using mixed valence Ce(III)/Ce(IV) as catalytic metal nodes, where the partial oxidation of Ce(III) in a mixed valent state caused the formation of Ce(III) and Ce(IV) redox couples with Ce(IV) performing OX-mimic activity [78]. Using a solvothermal process, Mao et al. (2022) synthesized an OX-mimic Cu(II)-MOF using 3-Amino-5-mercapto-1,2,4-triazole as an organic ligand [77]. The incorporation of the triazole unit Cu(II)-MOF showed excellent OX-mimic activity and initiated the TMB and ABTS oxidation in the absence of H_2_O_2_. In addition to this, Cu(II)-MOF showed strong antibacterial activity against *Escherichia coli* and *Staphylococcus aureus* [77]. While they do possess OX-mimic capabilities, these pristine MOFs exhibit poor catalytic activity and substrate specificity compared to natural oxidases. Therefore, synthesizing the MOF by stimulating the structural attributes of a natural enzyme would be a feasible strategy to address these limitations. In view of this, Li et al. (2020) synthesized a catechol oxidase-mimic MOF-NZ called MOF-818 by mimicking the active center (binuclear copper metal coordinated with six histidine molecules) of a catechol oxidase enzyme [76]. The developed MOF-818 exhibited excellent catechol oxidase-mimic activity and specifically oxidized 3,5-Di-tert-butylcatechol but not TMB and ABTS [76]. More such studies are important to increase the MOPF-NZ’s catalytic performance and substrate affinity.

#### 2.2.2. Modified MOF Nanozymes

**Figure 3:**
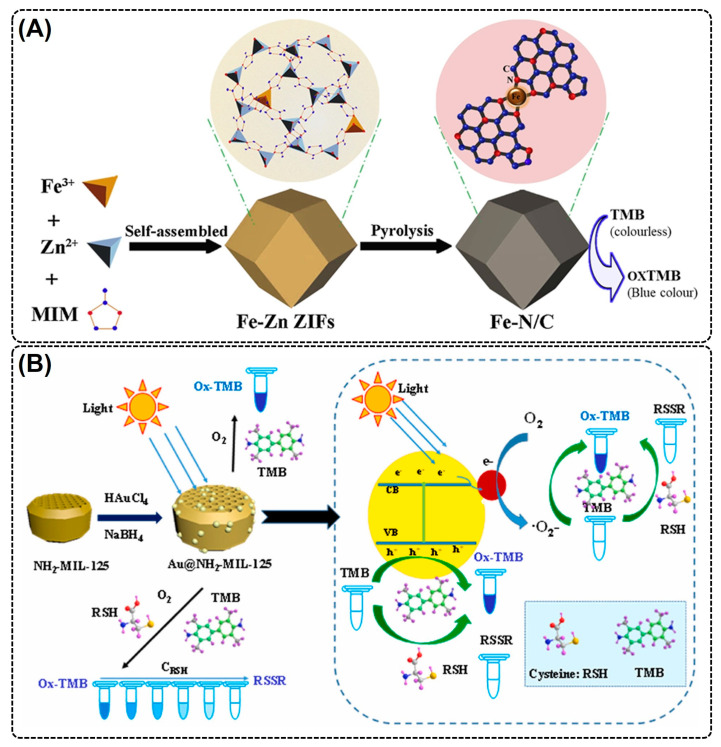
OX-mimic activity of MOF. Schematic illustration showing OX-mimic activity of (**A**) Fe-N/C ZIF MOF synthesized by precipitation and pyrolysis method. Reproduced with permission from [80] and (**B**) light-responsive AuNPs@NH_2_-MIL-125(Ti) MOF composite, wherein the AuNPs provided a high photocatalytic activity by photo-generated charge transfer and separation. Reproduced with permission from [81].

Similar to modified POX-mimic MOF NZs, different modification strategies have been employed to increase the catalytic performance of MOF-NZs by altering the structural properties of MOF-NZs. Using MOFs as a superficial template, Cao et al. (2018) developed OX-mimic CeO_2_ NPs by pyrolyzing Ce-MOF, wherein the Ce-metal ligands converted into ultrasmall CeO_2_ NPs (4 nm diameter), dispersing evenly on a porous carbonaceous framework [79]. Moreover, the pyrolysis created abundant oxygen vacancies in the CeO_2_ nanozyme, facilitating easy oxygen exchange and superior OX-mimic activity. In another study, Chen et al. (2020) developed an excellent OX-mimicking MOF containing size-controllable Fe-N/C nanozymes by precipitating 2-Methylimidazole, Fe(NO_3_)_3_·9H_2_O, and Zn(NO_3_)_2_·6H_2_O in methanol, followed by pyrolysis at 600–1000 °C to obtain a Fe-N/C catalyst [80]. The Fe-N/C catalyst serves as an electron acceptor and reduces subsequently adsorbed O_2_ molecules to form reactive oxygen species (ROS), which resulted in the oxidation of the TMB substrate to produce a blue color (Figure 3A). The Fe-N/C catalyst showed higher V_max_ and catalytic activity relative to Fe-N/C-CNT and free-Fe atoms; however, the catalyst showed lower substrate (TMB) affinity [80]. Interestingly, the catalytic activity varied significantly with pyrolysis temperature, pH, catalyst concentration, and catalyst particle size. Therefore, the pyrolysis of an MOF could be a feasible strategy to enhance their catalytic performance.

Many previous studies have deployed metal/metal oxide NP encapsulation and heteroatom doping strategies to increase the OX-mimic catalytic performance and stability of OX-mimic MOF-NZs. In one such study, Zhang et al. (2021) developed a light-responsive OX-mimic AuNPs@NH_2_-MIL-125(Ti) MOF-NZ by exploring the lower OX-mimic properties of Au NPs and a NH_2_-MIL-125(Ti) MOF [81]. The developed MOF showed high substrate affinity (~16-fold lower K_m_ value) and reaction velocity (~1.4-fold higher V_max_) for a TMB substrate compared to POX-mimic AuNPs@NH_2_-MIL-125(Ti). Moreover, the AuNPs on the NH_2_-MIL-125(Ti) facilitated photo-generated charge transfer and separation, leading to higher photocatalytic activity (Figure 3B). In another study, an OX-mimic Fe/Mn-MIL(53) MOF was developed by doping Mn ions [82]. The developed MOF showed higher catalytic activity due to increased electron transfer rate and O_2_·^-^ generation from multiple redox couples (Fe^2+^/Fe^3+^ and Mn^2+^/Mn^3+^). Moreover, the Fe/Mn-MIL-53 showed a 5-fold lower K_m_ value relative to Fe-MIL(53), exhibiting greater substrate affinity. Therefore, from the aforementioned studies, it is evident that an MOF modification with heteroatom doping and metal/metal oxide NPs could be a feasible strategy for attaining high OX-mimic activity; however, complex synthesis techniques and the requirement for toxic precursor molecules hinders their wide range of biomedical applications.

### 2.3. Superoxide Dismutase-Mimic

**Figure 4:**
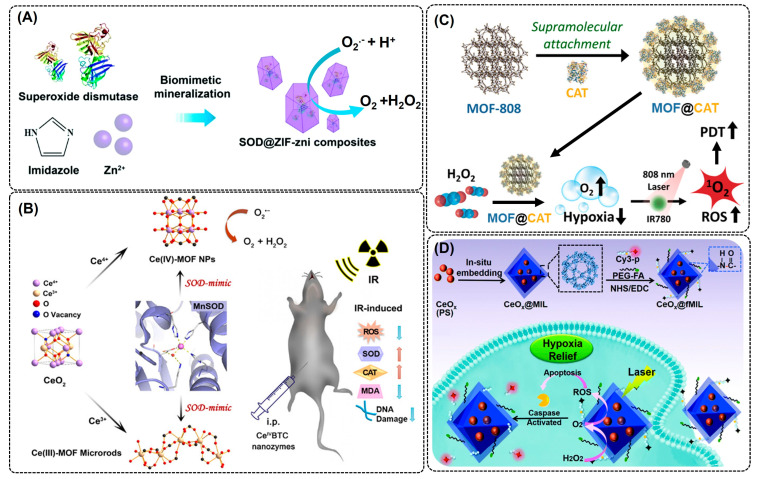
MOFs with SOD and CAT activity. Schematic illustration showing (**A**) SOD encapsulation in ZIF MOFs via biomimetic mineralization to enhance the catalytic activity, biocompatibility, and stability of enzyme. Reproduced with permission from [83]; (**B**) SOD-mimic Ce(III)- and Ce(IV)-based MOF nano/micro-composite synthesis for enhanced catalytic activity against ROS scavenging. Reproduced with permission from [87]; (**C**) direct CAT attachment to the surface of MOF-808 by using supramolecular interactions for designing CAT@MOF composite and its subsequent application in enhanced ROS scavenging leading to improved photodynamic therapy (PDT). Adapted with permission from [96]; and (**D**) synthesis of CAT-mimic MOF by in-situ embedding of CeO_x_ NPs. Adapted with permission from [104].

SOD is a crucial enzyme that carries out the simultaneous oxidation and reduction of unstable and toxic ROS, such as ^1^O_2_, O_2_·^−^ to H_2_O_2_, and is widely used for the treatment of ROS-induced health problems. However, the in vivo deployment of these enzymes has a few major challenges, including low storage stability, susceptibility to enzymatic aggregation and degradation, poor pharmacokinetics properties, and limited cellular uptake [83,84]. To overcome these challenges, immobilization or encapsulation into the porous matrix has been widely practiced. The encapsulation of SOD on porous MOFs using a biomimetic mineralization strategy increased the enzyme biocompatibility, stability, and activity, and is considered a promising platform for in vivo biomedical treatment of ROS-mediated health problems. Using a similar approach, Guo et al. (2022) immobilized SOD using a Zr-MOF as a precursor. The SOD@Zr-MOF showed an excellent ROS scavenging property that significantly reduced mitochondrial damage and cell death, and alleviated inflammation in the treated cells [83]. Bai et al. (2022) developed an SOD-encapsulated nanocomposite using zeolitic imidazole framework-zni (SOD@ZIF-zni) (Figure 4A) [85]. Compared to SOD, SOD@ZIF-zni showed high stability (temperature, pH, and storage), an in vitro anti-inflammatory effect, and therapeutic efficiency to treat inflammatory bowel disease [85]. Despite these merits, hybrid MOFs suffer from high synthesis costs and non-targeted action, which need to be addressed in the future. There are a few more reports which highlighted the SOD-mimic properties of MOFs and MOF composites, proving as an effective and excellent alternative to natural SOD for therapeutic applications [86,87,88]. A SOD-mimic cerium-based MOF using Ce(III) and Ce(IV) as an active site and 1,3,5-benzenetricarboxylic acid as an organic cross-linker was developed by Liu et al. (2022) for radioprotective applications against γ-radiation [87]. The Ce(IV)-MOF exhibited excellent SOD-mimic activity and demonstrated broad spectrum protection ability against γ-radiation by alleviating intracellular ROS and DNA damage without causing in vivo toxicity to exposed cells (Figure 4B) [87]. By mimicking the structural and functional attributes of natural SOD, Cu-tetrakis (4-carboxyphenyl) porphyrin MOF nanodots (Cu-TCPP-MOF NDs) were developed by coordinating Cu with N and O atoms [89]. The Cu-TCPP-MOF NDs showed excellent catalytic activity and efficiency as a ROS scavenger and catalyzed the enzymatic cascade reaction to convert H_2_O_2_ to H_2_O. The Cu-TCPP-MOF NDs showed a dose-dependent cellular uptake, cytoprotective ability, low cytotoxicity, and excellent pharmacokinetics and renal clearance compared to natural SOD or SOD @MOF.

### 2.4. Catalase-Mimic

Like SOD, catalase (CAT) is another important antioxidant enzyme that complements SOD activity to convert unstable toxic ^1^O_2_ or O_2_·^−^ to H_2_O and O_2_. Natural CAT and CAT-mimic nanozymes can be used in biomedical applications for preventing ROS-mediated cell membrane damage, treating inflammatory disorders, tumor cell growth inhibition [90,91], and environmental remediation (biodegradation of organic contaminants) [92]. Despite their promising application potential in ROS-based therapeutic applications, natural CAT suffers due to poor thermal stability and easy inactivation under harsh acidic tumor environments [93]. To address these limitations, a few studies immobilized CAT enzymes into an MOF and achieved high stability, reusability, and catalytic activity under adverse conditions (pH and temperature) [94,95]. Recently, Sim et al. (2023) developed a CAT@MOF-888 nanocarrier system by the direct interfacial conjugation of a CAT molecule into an MOF-888 surface for the intracellular uptake and transfer of CAT (Figure 4C). The MOF-888 enabled pH-responsive CAT detachment inside the cell and efficiently initiated ROS generation for photodynamic therapy of tumor cells [96]. Using the biomimetic mineralization encapsulation technique, Guo et al. (2021) immobilized CAT into ZIF-8 to achieve a highly stable and reusable CAT@ZIF8 MOF composite [97]. The resulting CAT@ZIF-8 exhibited exceptional catalytic activity, higher substrate (H_2_O_2_) affinity (K_m_-value for CAT is 63.4 mM and CAT@ZIF-8 is 16.1 mM), and thermal (5–75 °C) and pH stability (5–9) compared to the natural CAT enzyme. Similarly, Liang et al. (2019) and Zhang et al. (2019) observed similar-to-higher catalytic performance and stability (temperature, organic solvents, and denaturing agents) of CAT-immobilized MOFs compared to natural CAT enzymes [98,99]. However, enzyme immobilization is a cumbersome process and is only successful when the enzyme is compatible with the size of the MOF cavity. Also, the surface attachment via covalent and non-covalent interaction might expose enzymes to proteolytic degradation and leaching [100]. To address this, some of the catalase-mimic cerium oxide (CeOx) NPs have been explored for therapeutic applications [101,102]. However, their efficiency in vivo application was compromised under harsh acidic conditions [103]. To protect cerium oxide from losing its catalytic performance, Liu et al. (2021) embedded CeOx NP into the MIL-NH_2_ (CeOx@MIL) via a surface modification to enhance its catalytic activity under a harsh hypoxic environment (Figure 4D) [104]. The outer MOF shell protected inner-core CeOx NPs, as a result, a 9-fold higher apoptotic efficiency was observed with the CeOx-MOF compared to pristine CeOx NPs [104]. Despite CAT-mimic nanozymes being promising agents in anti-inflammation and cancer therapy, studies exploring their development and application have remained limited, which calls for extensive efforts in this area.

### 2.5. Multi-Enzyme Assemblies

Apart from the aforementioned examples of MOFs mimicking a single-enzyme activity, certain MOFs can display multi-enzyme activities under different operating conditions or sometimes even under similar conditions. In other cases, MOFs with enzyme-like activity are combined with natural enzymes (enzyme@MOFs) to obtain multi-enzyme assemblies. The ability of MOFs, alone or with an enzyme, to display two or more enzyme activities has made them appropriate tools for cascade catalytic reactions and therapeutics. The structural versatility and tunable properties of MOFs have made MOFs suitable candidates for enzyme immobilization. In view of this, extensive efforts have been made to harness the potential of MOF-NZs in enzyme immobilization. In one such report, a GOx enzyme was encapsulated into the highly porous POX-mimic Au/MOFs(Fe, Mn)/CNTs composite [105] and in another, POX-mimic 2D-BTC MOFs were used for the encapsulation [106]. In both cases, authors could achieve ultrasensitive and selective glucose detection from the clinical specimens while maintaining the extended stability and reusability of the enzyme. In addition to immobilizing single enzymes in MOFs, efforts have also been made towards developing multi-enzyme cascade systems by immobilizing dual enzymes. In a recent report, Hou et al. (2022) developed a highly stable and biocompatible SOD@CAT@MOF hybrid complex by exploiting ZIF-8 as an exoskeleton scaffold and MPEG_2000_-COOH for immobilizing SOD and CAT enzymes [84]. Interestingly, the developed MOF could not only demonstrate superior antioxidant properties for reducing H_2_O_2_ levels in simulated cell injury but also reduce ROS generation and enhance cell viability. It is worth noting that despite these merits, the encapsulated enzymes may exhibit reduced catalytic activity compared to their free counterparts and limited stability under ambient conditions, which calls for alternative approaches to develop multi-enzyme assemblies.

To this end, the incorporation of enzyme-mimicking metal or metal oxide NPs into the porous MOF construct has gained tremendous attention for achieving multi-enzyme-mimic assemblies that could potentially overcome the above-mentioned limitations. So far, various single and bimetal nanoparticles have been incorporated in POX-mimic MOFs to develop multi-enzyme mimic cascade complexes [107,108]. In a similar approach, Huang et al. (2017) combined the Gox-mimic Au NPs with POX-mimic Cu(II)-MOF nanosheets to develop a dual-enzyme mimic assembly and successfully exploited it for ultrasensitive glucose detection [107]. In another complex, intrinsic POX- and CAT-mimic Pt NPs were combined with a POX-mimic MOF(Fe) to develop dual-enzyme mimic MOFs [109]. Notably, the large surface area of MOFs allowed increased binding of Pt NPs and subsequently displayed more catalytic active sites. This in turn conferred the MOF complex with excellent catalytic activity and substrate affinity for H_2_O_2_ electrocatalysis than its pristine MOF counterparts.

Despite the merits of multi-enzyme mimic NP@MOF complexes, their requirement for tedious and challenging modification calls for facile alternatives to obtain complexes with desired properties. Prompted by this, Yang et al. (2022) fabricated a one-component multi-enzyme mimic Fe-PCN MOF by incorporating zirconium (Zr) ions as active metal nodes and Fe-porphyrin as an organic linker [110]. Interestingly, the developed MOF could exhibit superior intrinsic POX-, OX-, and phosphatase-like activities with Zr-O clusters providing active sites for both POX- and OX-mimics and Fe-centers for phosphatase-mimic activities. On account of this, the developed MOF could also demonstrate excellent sensitivity and substrate affinity for H_2_O_2_ detection under neutral conditions compared to other pristine MOFs and bimetallic systems. In another study, a mixed-valence Ce-MOF was designed by Luo et al. (2019) to mimic both POX and OX activities [111]. Notably, the developed MOF exhibited high substrate affinity (low K_m_) and catalytic activity compared to HRP. Despite emerging advancements in the developing multi-enzyme mimic systems, most efforts have been limited to POX- and OX-mimic systems. In order to truly meet the versatility of natural enzymes, extensive efforts are required to explore the other enzyme-mimic activities. Given this, Yang et al. (2021) synthesized a SOD- and POX-mimic MOF complex by employing PEG modification and Cu-Pd alloy incorporation in the MIL-101 MOFs (Cu-Pd@MIL-101) [112]. Reportedly, the developed MOF complex could effectively demonstrate ROS-mediated tumor therapy by releasing toxic ^•^OH radicals in the tumor microenvironment. While systems like these present themselves as promising alternatives to natural SOD- and CAT-based therapies, challenges associated with their biocompatibility and effective target site delivery need to be addressed to realize their practical feasibility in biomedical therapy.

## 3. Influential Factors and Strategies to Enhance MOF-NZs Activity

**Figure 5:**
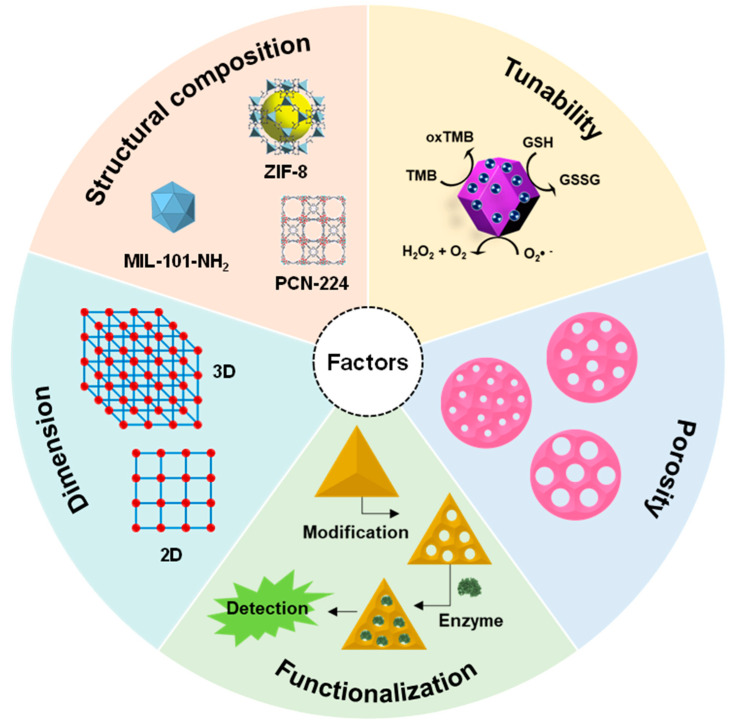
Major factors influencing the MOFs’ potential to act as nanozymes.

MOFs have been used to sense a wide range of analytes, including environmental waste, therapeutic molecules, and pathogens. Common factors that play a significant role in controlling the nanozyme activity of MOFs are discussed below and are also illustrated in Figure 5.

### 3.1. Structural Composition

The design and synthesis of MOFs rely on reticular chemistry. The combination of metal nodes and organic linkers allows for the creation of thousands of unique MOFs appealing to various applications [113]. Depending on the coordination, the metal ions (nodes) connect with the organic ligands termed secondary building units (SBUs). Here, the selection of an organic linker indicates the number of nodes that will be interconnected while SBUs dictate the node connectivity. Since the number of possible metal–linker combinations is countless, the design of an MOF implies a smart selection of the SBUs. This prompts the question of whether a new experimental or predicted structure contributes novel information. Modifying the geometry, length, ratio, and functional groups of the linker can tailor the size, shape, and internal surface characteristics of an MOF to suit a specific application [114]. Consequently, there is a need for a straightforward and applicable guideline to design MOF structures with specific desired properties. A common technique used for MOFs’ structural determination is single-crystal X-ray diffraction [115]. It is essential to elucidate their structure to continue this advancement. Machine learning and computational strategy can be used to predict the structure of MOFs, which helps to analyze their chemical diversity [116,117,118]. The building blocks (metal nodes and organic ligands) of MOFs and their structural similarity to natural enzymes have endowed them to mimic different enzymes. For example, Chen et al. (2019a) prepared a bionic ZIF-8 by incorporating 2-methyl imidazolate and central Zn^2+^ ions to mimic human carbonic anhydrase II (hCAII) [119]. The interlinking of coordinated central Zn^2+^ ions with imidazole formed a microporous network that resembled the geometry of the hCAII active site. The less imidazole-coordinated Zn^2+^ ions on the ZIF-8 surface acted as a Lewis acid and mimicked the reaction of natural enzymes, e.g., esterase and acetylcholinesterase, which breakdown ester into acid/alcohol and acetylcholine, respectively. In another report, Li et al. (2019) developed a porous coordination network (PCN-224) by using Zr_6_ clusters and tetrakis(4-carboxyphenyl)porphyrin (TCPP) [120]. They introduced Fe^3+^ in the core of porphyrin to mimic the catalytic active site of natural peroxidase and heme, which resulted in excellent POX-mimicking activity. Moreover, the POX-like activity of natural metalloporphyrin inspired the building of catalytic active MOFs [121]. Chen et al. (2020) took advantage of the metalloenzyme-like activity of Fe-MOF to detect glucose and H_2_O_2_ [122]. Its highly ordered structure demonstrated good POX-like activity where iron acts as a catalytic cofactor. In a study, Li et al. (2020) proposed MOF-818, where trinuclear copper atoms in the center mimicked natural catechol oxidase [76]. MOF-818 itself showed enzyme-mimic activity by oxidizing 3,5-Di-*tert*-butyl catechol (3,5-DTBC) efficiently with no POX-like activity. Qi et al. (2017) developed a luminescent MOF showing a POX-like catalysis [123]. The Tb^3+^-based luminescent MOF is only selective to H_2_O_2_ as the co-existence of interfering substances does not make a fluorescence enhancement. The advantage of using luminescent MOF-NZs prevents the use of natural enzymes as well as chromogenic substrates.

### 3.2. Tunability

The tunability of MOFs plays a pivotal role in designing materials with precise characteristics suited to particular tasks. This adaptability enables the customization of MOFs for applications such as catalysis and drug delivery [124,125]. The architectural tunability feature of MOFs inspires researchers to use them as nanozymes as well as carriers of natural enzymes, wherein MOFs encapsulate a natural enzyme through a coordination bond between metal ions and the carbonyl/oxygen-containing group of the protein. Wang et al. (2017) reported an artificial enzyme system where Ni-Pd hollow NPs, as well as glucose oxidase (GOx), were immobilized on the surface of ZIF-8 simultaneously via a co-precipitation method [126]. The resulting bio-composite exhibited the POX-mimic activity of hollow Ni-Pd NPs and retained the bioactivity of GOx. This multi-enzyme model had the potential to be used as a colorimetric and electrochemical glucose sensor. Liu et al. (2020a) prepared a “raisin pudding”-type ZIF-67/Cu_0.76_Co_2.24_O_4_ nanosphere by rationally modifying the weight ratio of ZIF-67 and Cu(NO_3_)_2_ [127]. This cobalt ion of the nanosphere allows it to mimic POX-, glutathione POX-, SOD-, and laccase-like activities. In essence, the tunability feature of MOFs underscores their versatility and potential biosensing applications, making them valuable materials for catalytic material design and application-specific requirements.

### 3.3. Porosity

The pore size of MOF-NZs greatly influences their catalytic activity. As most of the MOFs have nanoporous surfaces (pore size diameter 2 nm or smaller), large molecules cannot access their active sites. A series of one-dimensional (1D) mesoporous metalloporphyrin PCN-600 was prepared by Wang et al. (2014b); however, only PCN-600(Fe) demonstrated POX-mimic catalysis [128]. The better nanozyme performance of PCN-600(Fe) could be due to the large pore volume and even distribution of catalytic active sites. In another study, a stable, mesoporous MOF designed as PCN-222(Fe) was prepared using Fe-TCPP [121]. The large (about 3.7 nm in diameter) pore size of PCN-222(Fe) demonstrated excellent stability and oxidation of several substrates including pyrogallol, methyl benzidine, and o-phenylenediamine by mimicking a POX reaction in water. Apart from creating uniform porosity, MOFs can also be designed to exhibit varying pore sizes. In one such report, Lian et al. (2016) developed a porous PCN-888 containing three different pore sizes by the bi-enzyme coupling method [129]. While the large (~6.2 nm) and intermediate (~5 nm) pores encapsulated a single GOx molecule (150 kDa) and HRP molecule (44 kDa), respectively; the small (~2 nm) pores only mediated substrate diffusion. Interestingly, the developed MOFs could maintain excellent catalytic activity for several cycles and protect the encapsulated enzymes from trypsin digestion. Owing to the high reusability and stability of encapsulated enzymes, PCN-888 could also be employed for in vitro and in vivo applications.

### 3.4. Dimension

MOFs can exist in any of the three dimensions, which makes it imperative to study the influence of dimension on the catalytic activity of nanozymes. Jin et al. (2020) studied the OX-mimicking activity of 2D ZIF-67, wherein the large surface area of this dodecahedral MOF efficiently absorbs and oxidizes both chromogenic and fluorescent substrates without the participation of H_2_O_2_ [130]. Studies have shown that 2D MOFs exhibit better catalytic performance than 3D MOFs due to a larger surface area, rapid charge transfer, accessible active sites, and smaller barriers for substrate molecule diffusion [131,132,133]. Given this, an ultrathin 2D MOF (i.e., Cu-TCPP) was prepared by the solvothermal method and maintained the specific properties of each component. This nanosheet had excellent photothermal and photodynamic effects to utilize in biomedical applications [134]. The presence of the TCPP ligand in the framework architecture allowed singlet oxygen species production to kill the nearby cancer cells. In a study, Cheng et al. (2017) prepared 2D MOF nanosheets using binuclear metal clusters and TCPP ligands. The synthesized 2D MOF exhibited POX-like catalytic kinetics compared to bulky Zn-TCPP(Fe) [135]. It is recommended to develop more 2D MOF nanozymes with new favorable activity for biomedical applications.

### 3.5. Functionalization

Like other nanomaterials, the nanozyme activity of MOFs can also be tuned by surface modification. This offers MOFs to be used as multi-enzyme platforms via cascade reaction [21]. However, MOFs with larger pore sizes such as ZIF-8 and MIL-100(Fe) are suitable for these modifications. Post-synthesis modification offers better protection of the loaded enzymes by preventing leakage of immobilized enzymes. For instance, Zhao et al. (2020) synthesized boronic acid (BA)-functionalized hierarchically porous (HP) MIL-88B (i.e., GOx@HP-MIL-88B-BA), where BA offered direct immobilization of GOx and maintained the enzymatic activity [53]. Almost 86% of the enzymatic activity was retained after seven cycles due to low GOx leakage, suggesting the excellent reusability of Gox@HP-MIL-88B-BA. Moreover, numerous interfering substances were tested to check the selectivity of Gox@HP-MIL-88B-BA. The fabricated product is highly selective to detect glucose as no clear signal interference was observed even at highly concentrated maltose, lactose, and fructose. Moreover, the enzyme-loaded MOF exhibited a fast response to glucose (about 10 min) due to the enhanced mass transfer efficiency of the hierarchical porous structure of the MOF.

Several MOFs have demonstrated enzyme-mimic activity, but most fall short of matching the standards set by natural enzymes. Identified factors contributing to the poor nanozyme activity of MOFs include particle size, porosity, and the exposure of active sites. The catalytic efficacy of MOFs is closely linked to the distribution of pore sizes, emphasizing the need for sufficiently large pores to capture substrates. However, the location of catalytic active sites inside the material hampers catalysis performance.

To address these challenges, studies have explored various strategies, including the preparation of bimetallic MOFs, valence state regulation, and single-atom nanozymes, to enhance the catalytic performance of MOFs. Bimetallic MOFs exhibit improved catalytic performance, attributed to the potential difference between the two metals promoting electron transport and introducing defects in the structure, leading to more active sites [108,136,137]. However, understanding the interaction between the two metal nodes and the underlying mechanisms behind enhanced nanozyme activity requires further exploration. Another strategy involves the regulation of the chemical valence state of metal nodes, where the ratio of redox couple states plays a crucial role in determining catalytic activity [78,111]. Engineering the valence state can significantly enhance catalytic performance, as demonstrated by the development of a mixed-valence Ce-MOF with oxidase-like activity [78,83,138]. The regulation of valence state also offers potential applications in monitoring enzymatic activity. Additionally, the reduction of MOF particle size to the atomic level aims to maximize catalytic activity by increasing the ratio of exposed surface atoms [33]. The single-atom nanozymes (SAN) have shown promising results, such as a Fe-N-C SAN exhibiting excellent POX-mimicking activity and stability [139]. The developed SAN is used to detect a common nerve disease biomarker, butyrylcholinesterase (BchE). In another study, Zhao et al. (2019) demonstrated that heterogeneous single Fe atom catalysts with an active center like ‘heme’ in natural enzymes could mimic multiple enzyme-like activities including POX, OX, and CAT [140]. Therefore, the single atomization of MOF-NZs’ active sites preserves their intrinsic structural framework.

## 4. Applications of MOF-NZs

On account of the remarkable properties discussed in the earlier section, MOF-NZs have been exploited for extensive applications in diverse fields such as biosensing, therapeutics, antibacterials, and theranostics. Considering this, the present section has been compiled exclusively to discuss the application of MOF-NZs in the biosensing and therapeutic domains.

### 4.1. Biosensing Application

Biosensors are analytical devices that are used to detect the presence (qualitative) or concentration (quantitative) of biological/chemical analytes. A typical biosensor platform consists of two main components: a bioreceptor molecule (e.g., enzyme, antibody, aptamers, nucleic acids, etc.) that specifically interacts with the target analyte and a transducer element that converts the interaction of the bioreceptor with the target analyte into a quantifiable signal [141]. For the last 50 years, natural enzymes have been at the forefront of biosensors; however, their inherent shortcomings, such as poor stability under ambient conditions, complex synthesis, and tedious and expensive purification, limit their application. As per the World Health Organization’s ASSURED (Affordable, Sensitive, Specific, User-friendly, Rapid/Robust, Equipment-free, and Deliverable) criteria for on-site/point-of-care biosensor development [142], better alternatives are required to overcome these limitations. Given this, MOF-NZs have emerged as excellent alternatives in the biosensor platforms, and the following section briefly captures their application in colorimetric, fluorescence, and electrochemical biosensor development. These biosensors can be classified into four different categories based on their sensing strategies: (i) direct pristine MOF-NZ-based, (ii) natural enzyme encapsulating MOF-based, (iii) nanozyme activity modulation-based, and (iv) biological molecular recognition element (MRE)-based sensing.

#### 4.1.1. Colorimetric Biosensor

Colorimetric biosensors are simple, low-cost, user-friendly platforms that enable the naked-eye detection of target analytes. They work on the principle of natural enzyme/nanozyme-mediated catalysis of chromogenic substrates to form colored products. In the last decade, MOF-NZs have emerged as promising colorimetric transducers due to their versatile properties such as a high catalytic activity, presence of multiple active sites, high porosity, large surface area, and synthetic tunability. Owing to this, several MOF-NZ colorimetric platforms have been developed by employing the aforementioned sensing mechanisms. In the first strategy, pristine MOF-NZs are typically employed in place of natural enzymes like HRP to generate a colorimetric response, which is interpreted to monitor the presence of analytes. Numerous reports employing a similar strategy have been published for the detection of a wide range of analytes including H_2_O_2_ [143,144,145], small molecules [145,146,147], pesticides [82], and heavy metals [148,149,150]. In one such study, 2D Ni-based MOF (Ni-MOF) nanosheets, demonstrating excellent POX-mimic activity, were synthesized by using a one-step solvothermal method [143]. The developed MOFs exhibited high substrate affinity and could rapidly oxidize TMB in the presence of H_2_O_2_ to produce deep blue-colored products. The intensity of the colorimetric signal was found to be positively correlated with the analyte concentration, thereby allowing authors to detect H_2_O_2_ with high sensitivity (LOD = 8 nM) and precision. In another study, a similar strategy was employed to detect chromium ions (Cr (VI)) by replacing a POX-mimic MOF with an OX-mimic MOF. To do this, 3D 4-pyrazolecarboxylic acid-coordinated cupric MOFs (Cu-MOFs) were developed by a simple hydrothermal reaction, and incorporated into a paper-based device [149]. The working principle behind Cr (VI) ion detection relied on the electrostatic adsorption of Cr (VI) ions onto the MOF surface and the formation of a Cu-MOF@Cr(VI) complex, which could then trigger a redox reaction by efficiently interacting with the TMB substrate. Subsequently, a rapid (~3 min) detection of the Cr(VI) ion was achieved within the linear detection range of 0.5–50 μM. While the development of a portable biosensing platform is noteworthy, the sensitivity of the platform should be improved to meet the demands of on-site environmental monitoring.

**Figure 6:**
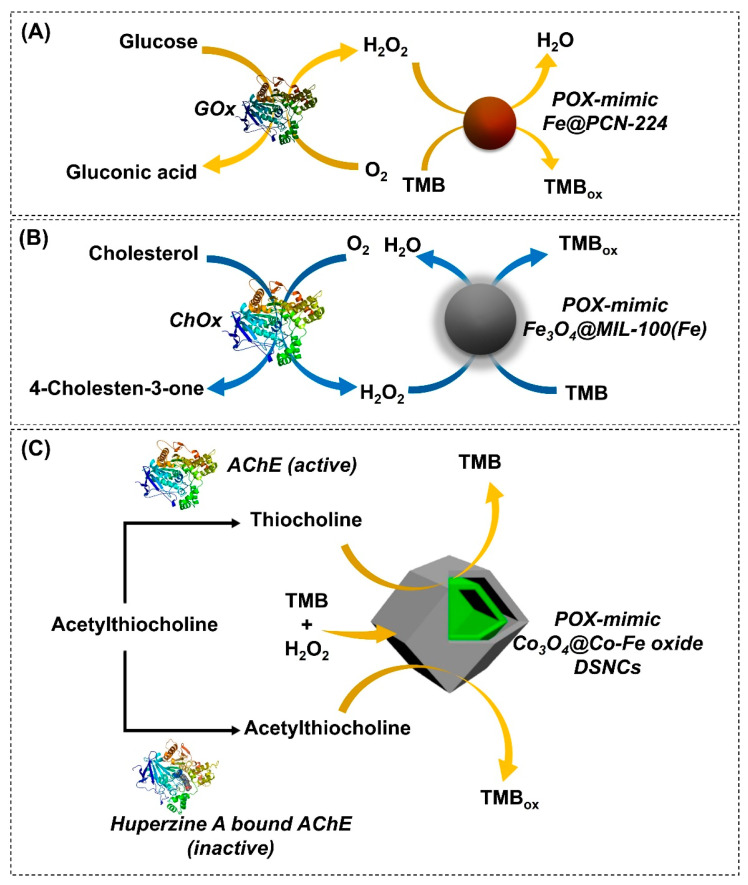
MOF-NZ-based colorimetric biosensors. Schematic illustration of (**A**) glucose and H_2_O_2_ detection by employing GOxFe@PCN224 MOFs. Adapted with permission from [120]; (**B**) cholesterol detection by employing ChOxFe_3_O_4_@MIL-100(Fe) MOFs. Adapted with permission from [61]; and (**C**) AChE activity detection in the absence and presence of huperzine A (AchE inhibitor) by utilizing Co_3_O_4_@Co-Fe oxide DSNC MOFs. Adapted with permission from [65].

Although pristine MOF-NZs have been widely exploited in myriad catalytic applications, their sensitivity and selectivity have remained rather low compared to their natural counterparts. On account of this, natural enzymes encapsulated in the porous MOFs (enzymes@MOFs) have emerged as promising candidates in catalysis due to the amalgamation of the intrinsic merits of enzymes (sensitivity, specificity, and selectivity) with that of MOFs (porosity and tunability). Such enzyme@MOFs form the basis for the second strategy in the biosensor platforms. So far, numerous enzyme@MOFs have been developed, with improved catalytic performance and a high stability and recyclability of the enzyme, by encapsulating natural enzymes, such as GOx, ChOx, and uricase, into the porous MOFs [151,152,153]. In one such report, Li et al. (2019) exploited the POX-mimic activity of a GOx@Fe(III)-functionalized PCN MOF to develop a biosensing platform for the colorimetric detection of glucose and H_2_O_2_ [120]. The immobilized GOx specifically oxidizes glucose molecules to release H_2_O_2_, which was then utilized by the POX-mimic MOF to oxidize TMB into a blue color product (TMB_ox_) (Figure 6A). The color intensity of TMB_ox_ is directly correlated with the target analyte concentrations. As a result, an ultrasensitive detection of glucose and H_2_O_2_ was obtained within a linear range (LR) of 30–800 µM and 2–100 µM, respectively. Furthermore, the developed platform offered a high sensitivity (LOD = 22 µM) and specificity toward glucose detection in the presence of other interference molecules such as fructose, lactose, and maltose. In another study, a simple colorimetric platform for cholesterol detection was developed by incorporating a natural ChOx enzyme into a POX-mimic MOF (ChOx@Fe_2_O_3_^−^ MOF) [61] to overcome the intrinsic limitations of conventional enzyme-based biosensors. The encapsulated ChOx enzyme oxidized cholesterol into H_2_O_2_, which was then utilized by the POX-mimic MOF to oxidize the TMB substrate using H_2_O_2_ as an electron acceptor (Figure 6B). An intense stable blue product (TMB_ox_) was generated, whose intensity was directly correlated with cholesterol concentration. As a result, authors could selectively detect serum cholesterol levels with a detection limit of 0.8 µM and an LR of 2–50 µM. In addition to that, the developed platform exhibited high selectivity and viability in the presence of other interfering molecules (glucose, urea, ascorbic acid, and amino acids) and spiked serum samples. In a similar study, a POX-mimic uricase@thorium-MOF (Th-MOF) was developed to detect UA concentration [154]. The sensing principle was based on the uricase-mediated oxidation of UA into allantoin and H_2_O_2_, which was subsequently used by the POX-mimic MOF to oxidize the TMB substrate using H_2_O_2_ as an electron acceptor. By employing the developed MOF-based sensing platform, researchers achieved sensitive detection of uric acid in an LR of 4–70 µM and an LOD of 1.15 µM. Moreover, the developed MOF-NZs offered excellent selectivity, stability, substrate affinity (towards H_2_O_2_), and practical feasibility when tested with spiked clinical samples in the presence of its other analogs and interfering molecules. From the aforementioned examples, it is evident that natural enzyme encapsulation in an MOF can aid in selective target detection without getting denatured. More reports on MOF-NZ-based biomarkers’ detection are discussed in Table 1.

In addition to exploiting enzyme@MOF-based nanozymes, researchers have also investigated alternative approaches for biosensor development, such as nanozyme activity inhibition, by employing biomolecules (DNA, thiocholine, GSH, AChE, and alkaline phosphatase) and their target analytes [155,156,157,158]. Cheng et al. (2022) developed a POX-mimic Ni(II)-doped bimetallic Fe-MOF (Ni_x_-Fe-MOF) for the colorimetric detection of H_2_O_2_ and GSH [64]. The developed MOF could detect H_2_O_2_ molecules with a detection limit of 0.59 µM and an LR of 1–80 µM. However, in the presence of GSH, the reduced thiol groups of GSH transformed the oxidized TMB product (blue) into colorless TMB, causing a decrease in colorimetric response. This decline in biosensor response indirectly correlated with GSH concentration to mediate its sensitive detection with an LOD of 1.88 µM and an LR of 10–400 µM. Furthermore, MOF-NZs offered highly selective GSH detection in the presence of other interfering molecules, such as glucose, sucrose, proline, dopamine, ascorbic acid, and metal ions. In another report, Chen et al. (2020b) developed a bimetallic Co_3_O_4_@CO-Fe oxide DSNC hybrid by combining the POX-mimic activity of Co_3_O_4_ and Fe oxide with ZIF-67 MOF for the ultra-sensitive and selective detection of H_2_O_2_ and acetylcholinesterase (AChE) activity [65]. The remarkable POX-mimic activity of the inner Co_3_O_4_ and outer CO-Fe oxide shell synergistically decomposed H_2_O_2_ to form ^•^OH radicals, which oxidize TMB to form a stable blue product. As a result, a visual response corresponding to H_2_O_2_ concentration was observed within an LR of 0.2–600 µM, enabling sensitive H_2_O_2_ detection (LOD = 20 nM). Furthermore, researchers could also detect AChE activity by inhibiting the POX-like activity of the developed MOF. This inhibition was induced by thiocholine, a product of the AChE-mediated hydrolysis of acetylthiocholine (Figure 6C). As a result, a decline in the TMB_ox_ signal was observed, which indirectly correlated with AChE activity. The hybrid MOF could detect AChE activity with high sensitivity (LOD = 2 × 10^−4^ mU mL^−1^) in the linear detection range between 8 × 10^−4^ mU mL^−1^ and 1 mU mL^−1^. It is worthwhile to note that the high POX-mimic activity of the MOF can aid in the sensitive detection of thiol-containing biomolecules; however, their specificity and selectivity are often compromised in complex biological matrices.

To overcome these limitations, the use of molecular recognition elements (MREs), such as antibodies, aptamers, nucleic acids, and enzymes, that can specifically interact with the target analyte are actively being explored. Recently, these MREs showed promising potential for designing sensing platforms with high sensitivity, specificity, and practical viability in real sample analysis. Under this scope, Xu et al. (2021) developed a simple MOF-NZ-linked immunosorbent assay (MOF-LISA) for the highly sensitive detection of aflatoxin B1 (AFB1) [159]. To do this, they immobilized a secondary antibody into POX-mimic MIL-88 MOF by covalent coupling. The sensing of AFB1 was achieved by its inhibitory effect of AFB1 on POX-mimic activity, causing a decline in colorimetric response. As a result, the developed platform achieved a sensitive detection of AFB1 (LOD = 0.009 ng mL^−1^) with high sensitivity (~30%) and specificity (~7%) comparable to that of conventional ELISA. Over these advantages, the poor stability, high cost of synthesis, and susceptibility to easy denaturation limit the application of antibody-based biosensors in in-field clinical diagnosis. Recently, aptamers have emerged as excellent surrogates for antibody-based sensors due to their high stability, low-cost synthesis, and remarkable specificity. Aptamers are short single-stranded DNA or RNA molecules that can specifically interact with various molecules such as proteins, nucleic acids, and microbes. Owing to the aforementioned merits, aptamers have been successfully employed in developing biosensor platforms [15,160]. Dang and Zhao (2020) developed a simple colorimetric biosensing platform by using an aptamer immobilized POX-mimic MOF composite (aptamer@Au/MOFs(Fe, Mn)/CNTs) for sulfadimethoxine (SDM) detection [68]. In the absence of an SDM molecule, the POX-mimic activity of the bimetallic MOF was inhibited by aptamer binding, preventing TMB oxidation. However, in the presence of SDM, the aptamers left the MOF surface and exposed the active catalytic sites of the MOF, thereby recovering the MOF’s POX-mimic activity. As a result, oxidized TMB (blue) was formed, which mediated SDM detection within an LR of 0.54–41.58 µg L^−1^ and an LOD of 0.35 µg L^−1^. In addition, the MOF-NZ also exhibited high selectivity in the presence of other antibiotics, such as sulfathiazole, sulfamonomethoxine, sulfamethoxazole, and oxytetracycline, and showed estimated recoveries in the range of 86 to 127% in tap water samples. In summary, the functionalization of MOF-NZs with target-specific MREs, antibodies, or aptamers could aid in the detection of target analytes with high sensitivity and specificity from the clinical specimens. However, the high synthesis cost, limited availability of aptamers, and complex synthesis techniques are the major challenges limiting their applications.

#### 4.1.2. Fluorescence Biosensors

**Figure 7:**
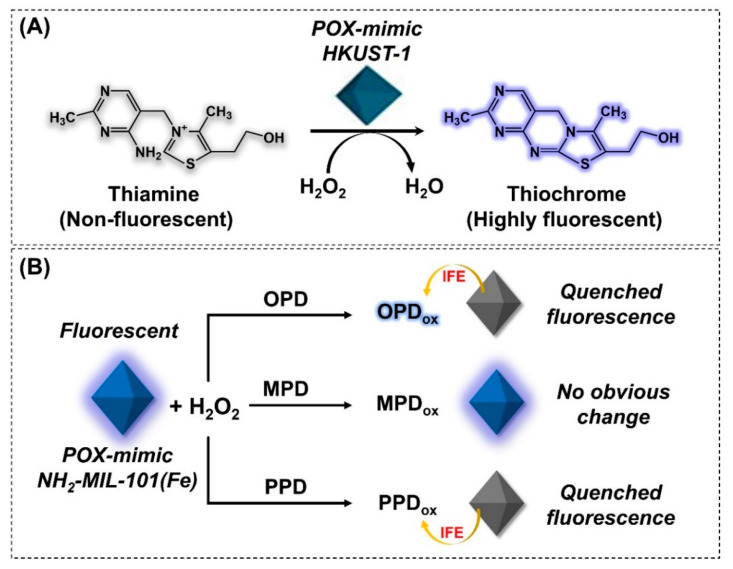
MOF-NZ-based fluorescence biosensors. Schematic illustration of (**A**) thiamine detection by utilizing HKUST-1 MOFs. Adapted with permission from [165] and (**B**) detection and differentiation of o-pheyldiamine (OPD) from other aromatic amines (e.g., p-phenylenediamine (PPD) and m-phenylenediamine (MPD)) using POX-mimic NH_2_-MIL-101(Fe). Adapted with permission from [163].

Fluorescence-based biosensors are another type of optical sensor that mediate the qualitative and quantitative detection of target analytes. Generally, these biosensors are simple, rapid, and easy-to-use platforms that mediate target molecule detection with high sensitivity. To date, several nanozymes, including metal and metal oxide NPs, quantum dots (QDs), and graphene, have been used to develop fluorescence-based biosensors [161]. However, their high cost, poor stability, low catalytic activity, and toxicity call for better alternatives. Given this, several MOF-NZs exhibiting outstanding properties, such as high catalytic activity, prolonged stability, low cost, ease of tunability, and biocompatibility, have been developed [162,163,164]. For example, by employing pristine MOF-NZs, Tan et al. (2015) developed a fluorescence-based biosensor by incorporating POX-mimic Cu(II)-MOF (HKUST-1) for thiamine detection [165]. Under alkaline conditions (pH 11.0), HKUST-1 used H_2_O_2_ to oxidize non-fluorescent thiamine to highly fluorescent thiochrome (Figure 7A). The intensity of the fluorescence signal is directly correlated with thiamine concentration in the LR of 4–700 µM. Moreover, by exploiting the developed platform, authors could achieve a detection limit of 1 µM for thiamine detection, which is nearly 50-fold lower than that of the HRP-based fluorescence assay. Reportedly, certain MOFs can exhibit intrinsic fluorescence properties, which could be exploited in fluorescence biosensors to overcome the photo-bleaching effect of fluorescent tags. In line with this, Xia et al. (2022) developed a ratiometric fluorescence-based detection platform by using POX-mimic NH_2_-MIL-101(Fe) for the detection and differentiation of OPD and its isomers [163]. Apart from exhibiting intrinsic POX-mimic activity, the NH_2_-MIL-101(Fe) showed intrinsic fluorescence properties (emission at 445 nm) under acidic conditions (pH = 5.0), which together formed the basis for OPD detection and differentiation. The unsaturated Fe-O clusters in MOF-NZs utilized H_2_O_2_ molecules to oxidize a non-fluorescent OPD substrate into a fluorescent product (OPD_ox_) (emissions at 574 nm), thereby quenching the inherent fluorescence of MOF-NZ. The changes in the ratio between fluorescence emission signal readouts were quantified to detect OPD concentration and differentiate between different phenylenediamine isomers (Figure 7B). The developed sensor probe could detect OPD with an LOD of 1.5 µM and an LR of 5–1200 µM. In another study, Hou et al. (2020) exploited a similar MOF, NH_2_-MIL-101(Fe), with intrinsic fluorescence (pH = 5.0) and nanozyme activity to design a rapid, sensitive, and selective fluorescence-based platform for catechol detection [166]. The POX-mimic property of NH_2_-MIL-101(Fe) reduced the H_2_O_2_ to form ^•^OH radicals, which in turn triggered the oxidation of catechol to form 1,2-benzoquinone. Subsequently, the as-produced 1,2-benzoquinone interacted with the amino group of the MOF by Michael addition reaction and quenched its inherent fluorescence signal. This decrease in fluorescence signal intensity indirectly correlated with catechol concentration, which resulted in the detection of catechol with high sensitivity (LOD = 0.0913 µM) and specificity. While it is worth noting the excellent performance of the aforementioned MOFs in fluorescence biosensor development, their requirement to operate in acidic conditions (pH = 5.0) may be less than ideal for clinical sample analysis. As a result, novel MOFs, exhibiting excellent catalytic activity and stability under neutral or close to neutral conditions, need to be developed.

Besides pristine MOF-NZ-based biosensors for direct analyte detection, researchers have also exploited natural enzyme-encapsulating MOFs in fluorescent biosensor development. Notably, some of these MOFs are synthesized by using fluorogenic ligands (e.g., terephthalic acid—TA), which generate fluorescence signals on oxidation. Hassandzadeh et al. (2018) used ChOx immobilized MOF (ChOx@Zn-TA-MOF) and POX-mimic silver-nanocluster-decorated molybdenum disulfide nanosheets (Ag-MoS_2_-NS) for sensitive cholesterol detection [167]. The encapsulated ChOx enzyme oxidized cholesterol to release H_2_O_2_, which was later utilized by POX-mimic Ag-MoS_2_-NS to oxidize fluorescent TA ligands, forming a fluorescent 2-hydroxyterephthalic acid (emission at 445 nm). The fluorescence intensity was directly correlated with cholesterol concentration in the LR of 0.06–15 µM, with excellent sensitivity (LOD = 30 nM) and specificity. In another study, Lin et al. (2018) immobilized GOx with POX-mimic MIL-53(Fe) to develop a label-free, sensitive, and stable glucose-sensing platform [168]. The encapsulated GOx enzyme catalyzes the oxidation of glucose molecules to form gluconic acid and H_2_O_2_. The POX-mimic MOF subsequently reduced H_2_O_2_ molecules to form ^•^OH radicals, which in turn oxidized the TA organic ligands to generate fluorescent signals. The developed sensing platform detected glucose in an LR of 0.5–27 µM and an LOD of 8.44 nM.

Apart from exploiting enzyme@MOFs in fluorescence biosensors, researchers have also investigated the nanozyme activity modulation strategy by employing target specific-aptamer molecules [169,170,171]. In one such study, Ali et al. (2022) utilized intrinsically fluorescent POX-mimic Cu-MOFs to develop a label-free fluorescence biosensor platform for thrombin detection [169]. The proposed sensor relied on a simple fluorescence turn-off/turn-on mechanism mediated by thrombin-specific aptamers. In brief, the thrombin-specific aptamers could adsorb onto the Cu-MOFs surface and quench/inhibit the intrinsic fluorescence and POX-like activity of the MOFs by a surface passivation mechanism causing the sensor to be in an ‘off-state’. However, in the presence of thrombin molecules, the aptamers desorbed from the Cu-MOF surface due to their high binding affinity towards the target analyte. Consequently, the fluorescence and the POX-mimic activity of Cu-MOF were restored, rendering the sensor to be in a ‘turn-on’ state. The recovery in MOFs’ fluorescence/POX-mimic activity was positively correlated with thrombin concentration, allowing authors to obtain an LOD of 110 fM. Interestingly, the recovery of POX-mimic activity was exploited to mediate the colorimetric detection of thrombin by oxidizing colorless POX substrates (TMB + H_2_O_2_) into the blue-colored product. As seen in the case of fluorescence recovery, the intensity of the colored product was also found to be positively correlated with analyte concentration, thereby resulting in an LOD of 350 pM. More examples of MOF-NZ-based fluorescence sensors have been captured in Table 1.

#### 4.1.3. Electrochemical Biosensors

**Figure 8:**
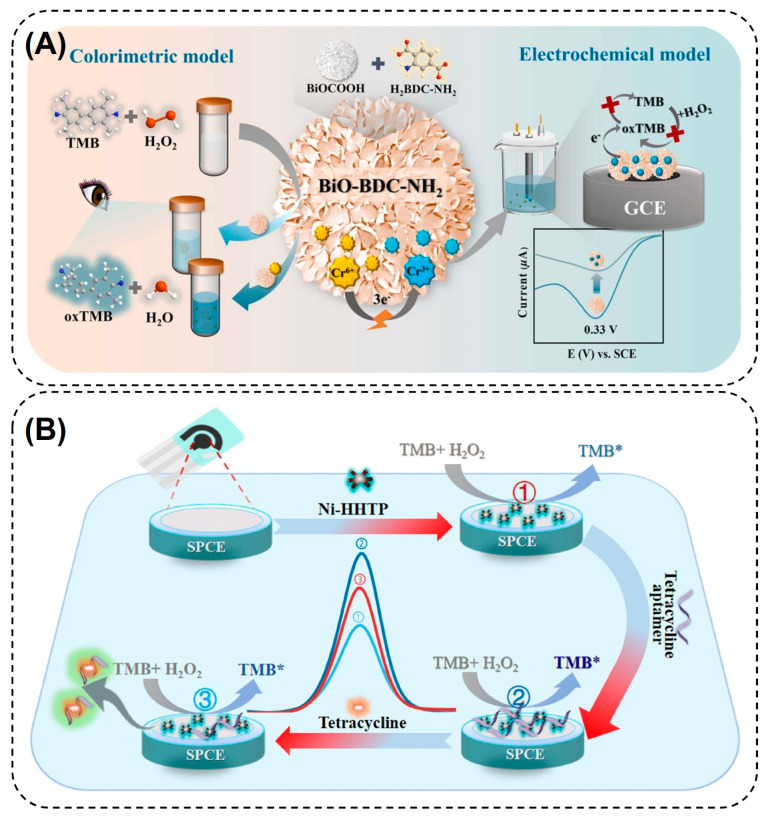
MOF-NZ-based electrochemical biosensor. Schematic illustration of the electrochemical (**A**) and colorimetric Cr(VI) ion detection by using POX-mimic BiO-BDC-NH_2_ MOF. Reproduced with permission from [182] and (**B**) tetracycline residue detection by using POX-mimic Ni-HHTP MOF, (TMB*: oxidized TMB). Reproduced with permission from [186].

Electrochemical (EC) biosensors are analytical devices that work on transducing biochemical events into electrical signals [172]. Owing to their high sensitivity, rapid response, ease of operation, and miniaturization, these biosensors have seen promising developments in fields including disease monitoring, food safety and quality testing, and environmental surveillance [173]. Despite these merits, EC biosensors suffer from several limitations, such as the need for redox-active analytes, poor selectivity, reusability, and signal reduction due to interfering molecules and electrode fouling, which calls for the development of better platforms [174]. In recent years, several strategies have been developed to improve the signal amplification and analytical performance of EC biosensors. Under this scope, pristine MOFs and their composites have been incorporated into different EC biosensing platforms to detect various target molecules including H_2_O_2_ [175,176], small molecules [177,178,179,180], enzyme activity [105], disease biomarkers [172], and environmental pollutants [181]. In one such study, Lu et al. (2020) developed a sandwich-hybrid nanocomposite by incorporating Au nanofibers (AuNFs) and MoS_2_ nanosheets into the Fe_3_O_4_-MOFs (AuNF@ Fe_3_O_4_-MOF/ MoS_2_) to design an EC sensing platform for H_2_O_2_ detection [175]. The incorporation of AuNFs and MoS_2_ nanosheets into the MOF framework not only increased the electrical conductivity of MOFs but also enhanced their catalytic performance and stability under harsh conditions. Owing to this, a sensitive detection of H_2_O_2_ with an LOD of 0.9 µM and a wide LR of 5 µM–120 mM could be achieved in the proposed assay. While it is noteworthy to see that the MOFs and their composites, on their own, can mediate EC analyte detection due to electroactivity, they can also enable colorimetric detection by mimicking enzymatic activity. Given the ability of MOFs to effectively combine the merits of colorimetric and EC modes in one platform, the development of dual-mode biosensors is slowly gaining due attention. To this end, Yang et al. (2023) incorporated a POX-mimic 3D bismuth oxide MOF (BiO-BDC-NH_2_) in a colorimetric/EC dual-mode biosensor for Cr(VI) ion detection (Figure 8A) [182]. In the single colorimetric mode, Cr(VI) ions could significantly increase the POX-mimic activity of MOFs and rapidly oxidize the TMB substrate to form a deep blue product by using H_2_O_2_. However, in the dual mode sensor, the Cr(VI) ions were electrochemically reduced into Cr(III), which significantly inhibited the POX-like activity of the MOFs. As a consequence, the oxidation of the TMB substrate was drastically reduced, resulting in the appearance of a faint blue color. The intensity of the blue color was inversely correlated with the Cr(IV) ion concentration. This change was electrochemically interpreted by electrochemical impedance spectroscopy to obtain an LOD of 9 pg mL^−1^.

The detection and quantification of disease biomarkers using molecular diagnostics tools are still challenging due to their need for established laboratories, costly equipment, trained professionals, long turnaround time, and lack of POC setup. Owing to this, better techniques need to be developed, which can not only overcome these limitations but also display high sensitivity. Prompted by this need, MOF-NZs incorporating biological MREs (aptamers, DNAzymes, antibodies, etc.) have been exploited in biosensor platforms for the rapid EC detection of various disease biomarkers [176,183]. Li et al. (2018) designed a Pd NP-decorated POX-mimic composite MOF (PdNPs@Fe-MIL88-NH_2_) for the ultrasensitive detection of miR-122 (a biomarker for drug-induced liver injury) using electrical impedance spectroscopy (EIS) and differential pulse voltammetry (DPV) techniques [184]. The high POX-mimic activity of PdNPs@Fe-MIL88-NH_2_ resulted in the highest catalytic oxidation of TMB in the presence of H_2_O_2_. The PdNPs@Fe-MIL88-NH_2_ nanohybrids assisted with target-catalyzed hairpin assembly and resulted in an increased electron transfer resistance with a logarithmic increase of miR-122 concentration. By employing the developed sensor, miR-122 was detected with an LOD of 0.003 fM and a wide linear detection range of 0.01fM to 10 pM. Using the DPV technique, Cao et al. (2022) developed an aptasensor for detecting a prostate cancer antigen (PSA) using POX-mimic PtNPs@Co_3_O_4_ biofunctionalized with PSA aptamer2 (PtNPs@Co_3_O_4_-*Apt2*) and magnetic NPs functionalized with PSA aptamer1 (MNPs-*Apt1*) [185]. In the presence of PCA, the PtNPs@Co_3_O_4_-*Apt2* and MNPs-*Apt1* formed a sandwich structure, resulting in the catalytic oxidation of TMB in the presence of H_2_O_2_. As a result, the electric charge transfer increased corresponding to PSA concentration. The PtNPs@Co_3_O_4_-*Apt2* coupled with MNPs-*Apt1* showed sensitive detection and magnetic separation of the sandwich structure, with a linear PSA detection range of 0.01–10 ng L^−1^ and an LOD of 0.0079 ng L^−1^. Therefore, these studies show that the MOFs and their composites with various nanozymes and MREs are the most promising POC platforms for accurate and very low-level detection of various disease biomarkers (Table 1), which can be deployed for detecting early-stage outbreaks and guiding proper clinical therapeutics.

Apart from detecting disease biomarkers, biological MRE-incorporating MOFs have also been exploited for the EC detection of environmental/food contaminants [186,187,188]. A portable EC biosensor was developed for tetracycline residue detection by incorporating POX-mimic hexahydroxytriphenylene-coordinated nickel (Ni-HHTP) MOFs onto a screen-printed electrode and employing square-wave voltammetry (Figure 8B). The working principle behind tetracycline detection was based on a turn-on/turn-off mechanism, wherein the non-covalent adsorption of tetracycline-specific aptamers onto the MOF surface significantly enhanced its POX-like activity. Consequently, more TMB substrates could be oxidized in the presence of H_2_O_2_ to form a deep blue product and the sensor was stated to be in an ‘on-state’. However, in the presence of tetracycline residues, the aptamer molecules desorbed from the MOF surface owing to their high binding affinity towards the analyte. As a result, the catalysis of TMB/H_2_O_2_ and the electrochemical signal were significantly reduced, and the sensor was confirmed to be in an ‘off-state’. Based on this strategy, authors could successfully analyze tetracycline residues with a detection limit of 1.9 pM and an LR of 10 pM–1 μM. Furthermore, the developed biosensor could also detect the tetracycline residues in meat and milk samples with estimated recoveries ranging from 94 to 108%, affirming their promising potential in food safety monitoring. More examples of MOF-NZ-based EC sensors have been captured in Table 1.

**Table 1:** MOF-NZ-based colorimetric, fluorescence, and electrochemical biosensors.

Enzyme-Mimic Type	Synthesis Method	Catalytic Ligand/NPs	Organic Linker	Substrates Used	Target Analyte	Linear Range	Limit of Detection	Reference
**Colorimetric-based Sensors**								
Peroxidase	Hydrothermal	Fe(III)	Fumaric acid	TMB, H_2_O_2_	Thrombin	10–80 nM	0.8 nM	[189]
	Hydrothermal	Fe(III), GO	2-aminoterephthalic acid	TMB, H_2_O_2_	Benzo[a]pyrene-7,8-diol-9,10-epoxide DNA adduct	0.2–100 ng mL^−1^	0.268 ng mL^−1^	[190]
	Self-assembly strategy	Cu(II)	4,4′-bipyridine	ABTS, H_2_O_2_	Alkaline phosphatase	1–34 U L^−1^	0.19U L^−1^	[62]
	Self-assembly strategy	Fe_2_O_3_ NPs, Fe(III)	1,3,5-benzene tricarboxylic acid	TMB, H_2_O_2_	Cholesterol	2–50 µM	0.8 µM	[61]
	Direct mixing	Fe(II)	tris(p-amino phenyl)amine	DMB, H_2_O_2_	Glutathione	0.02–20 mg L^−1^	3.25 nM	[191]
	Self-assembly strategy	Pt NPs	2-amino terephthalic acid	TMB, H_2_O_2_	Hg(II)	0–10 nM	0.35 nM	[66]
	Solvothermal process, H_2_-reduction methods	Ni(II), Fe(III)/Fe(II)	2-amino terephthalic acid	TMB, H_2_O_2_	H_2_O_2_	1–80 µM	0.59 µM	[64]
					Glutathione	10–400 µM	1.88 µM	
	Solvothermal process, chemical reduction	Au NPs	2-amino terephthalic acid	TMB, H_2_O_2_	Cysteine	1–10 µM	0.14 µM	[67]
					H_2_O_2_	2–10 µM	0.24 µM	
	Precipitation method, chemical reduction	Pt NPs	*N*,*N*’-dimethylformamide	TMB, H_2_O_2_	Dopamine	1–60 µM	0.42 µM	[192]
	Pyrolysis	Fe(III)	1,4,5,8-naphthalene tetracarboxylic dianhydride	TMB, H_2_O_2_	Alkaline phosphatase	0.05–6.00 U L^−1^	0.03 U L^−1^	[193]
	Precipitation method	Cu(II)	Hexamine	TMB, H_2_O_2_	Dopamine	0.5–3.5 mM	4.2 µM	[194]
	Solvothermal method	Fe(III)	*N*,*N*’-dimethylformamide	TMB, H_2_O_2_	Salicylic acid	0.4–28 µM	0.26 µM	[44]
	Solvent-free method	Carbon dots (CDs), Cu(II)	2-methylimidazole	TMB, H_2_O_2_	Glutathione	0–40 µM	0.15 µM	[195]
	Precipitation method	Fe(III)	Terephthalic acid	TMB, H_2_O_2_	Deoxyribonuclease I	0.2–7.0 U mL^−1^	0.09 U mL^−1^	[16]
	Air oxidation	Cu(II)	4,5-dicyanoimidazole	TMB, H_2_O_2_	H_2_S	0.6–30 μM	0.071 μM	[196]
	Hydrothermal	Fe(III)	Polyoxyethylene-polyoxypropylene	TMB, H_2_O_2_	Aflatoxin B1	0.01–20 ng mL^−1^	0.009 ng mL^−1^	[159]
Oxidase	Solvothermal process, chemical reduction	Au NPs,	2-amino terephthalic acid	TMB	Cysteine	1–10 µM	0.15 µM	[81]
	Precipitation method, pyrolysis	Fe(III)	2-methylimidazole	TMB	Alkaline phosphatase	0.05–100 U L^−1^	0.02 U L^−1^	[80]
	Precipitation method	Co NPs	2-methylimidazole	TMB	Glutathione	0.05–30 µM	36 nM	[51]
	Precipitation method	Ce(IV)/Ce(III)	1,3,5-benzene tricarboxylic acid	TMB	Hg(II)	0.05–6 µM	10.5 nM	[197]
	Precipitation method, and pyrolysis	Co NPs, AuAg nanoclusters, and CNT	2-methylimidazole	TMB	Ochratoxin A	0.001–10 µg L^−1^	0.21 ng L^−1^	[198]
Multi-enzyme assembly	One-step carbonization	Ce(IV)/Ce(III), GOx	Trimesic acid	TMB, H_2_O_2_	H_2_O_2_	0.5–100 µM	0.42 µM	[105]
					Glucose	1–100 µM	0.69 µM	
	Microwave heating	CoNPs, GOx	2-amino terephthalic acid	TMB, H_2_O_2_	Glucose	0.25–30 µM	156 nM	[199]
	Solvothermal process	Fe(III), GOx	Meso-tetra(4-carboxyphenyl)porphine	ABTS, H_2_O_2_	Glucose	0.5–100 µM	0.28 µM	[200]
	Precipitation method	Fe-polydopamine, GOx	2-methylimidazole	TMB, H_2_O_2_	Glucose	5–100 µM	1.1 µM	[201]
	Cation substitution strategy	2D-Fe-BTC, GOx	1,3,5-benzene tricarboxylic acid (BTC)	TMB, H_2_O_2_	H_2_O_2_	0.03–40 µM	36 nM	[106]
					Glucose	0.04–20 µM	39 nM	
	Calcination	Mn_3_O_4_ NPs, ChOx	2-methylimidazole	TMB, H_2_O_2_	Cholesterol	40–500 µM	4.85 µM	[202]
	Solvothermal process	Cu(II), Au NPs	Trifluoro acetic acid	TMB, H_2_O_2_	Glucose	10–400 µM	8.5 µM	[107]
**Fluorescence-based sensors**								
Peroxidase	Solvothermal process	Fe(III)	2-amino terephthalic acid	OPD, H_2_O_2_	o-phenylenediamine	5–1000 µM	1.0 µM	[163]
	Solvothermal process	Fe(III)	2-amino terephthalic acid	Amplex red, H_2_O_2_	Choline	0.5–10 µM	27 nM	[203]
					Acetylcholine	0.1–10 µM	36 nM	
	Hydrothermal process	Fe(III)	2-amino terephthalic acid	Terephthalic acid, H_2_O_2_	Choline	0.1–10 µM	20 nM	[204]
					Acetylcholine	0.01–100 µM	8.9 nM	
Multi-enzyme assembly	Amidation coupling reaction	Fe(III), GOx	2-amino terephthalic acid	TMB, H_2_O_2_	Glucose	0.1–600 μM	0.0428 µM	[205]
Oxidase	Precipitation method, and pyrolysis	Co NPs, AuAg nanoclusters, and CNT	2-methylimidazole	TMB	Ochratoxin A	0.001–10 µg L^−1^	0.17 ng L^−1^	[198]
**Electrochemical sensors**								
Peroxidase	Solvothermal process	Pt NPs, FeTCPP	Fe(III)meso-5,10,15,20-tetrakis(4-carboxyphenyl)porphyrin chloride (FeTCPP)	H_2_O_2_	Telomerase activity	50–5 × 10^5^ Hela cells mL^−1^	20 Hela cells mL^−1^	[109]
Multi-enzyme assembly	Solvothermal process	FeTCPP	Fe(III)meso-5,10,15,20-tetrakis(4-carboxyphenyl)porphyrin chloride (FeTCPP)	H_2_O_2_	H_2_O_2_	3–100 µM	1.1 µM	[206]
				NaNO_2_	NO	5–200 µM	1.3 µM	
Peroxidase	Hydrothermal process	Cu@Au, Fe_3_O_4_	2-amino terephthalic acid	TMB, H_2_O_2_	Cardiac troponin I	0.5–100 ng mL^−1^	16 pg mL^−1^	[207]
Peroxidase/Oxidase	Solvothermal process	Fe(III), Au NPs	Terephthalic acid	TMB, H_2_O_2_	miR-721 (acute myocarditis biomarker)	0.5 fM–1 nM	0.25 fM	[208]
Peroxidase	Hydrothermal process	Pt NPs	2-amino terephthalic acid	TMB, H_2_O_2_	Exosomal miRNA	1 fM–1 nM	2.00 fM	[209]

### 4.2. Therapeutic Applications of MOF-NZs

MOFs have gained recognition not only as advanced functional nanomaterials in materials science but also in the field of biomedicine. In recent years, there has been a growing number of publications showcasing the therapeutic potential of MOF-NZ systems [46,57,210]. This can be attributed to the promising characteristics of MOFs, such as their biocompatibility, ability to tune chemical composition, and versatile catalytic properties [24,211]. These attributes make MOFs an appealing tool for various therapeutic applications, including chemodynamic, photodynamic, photothermal, anti-inflammatory, and antibacterial therapies. A summary of the therapeutic applications of MOF-NZ systems can be found in Table 2.

#### 4.2.1. Chemodynamic Therapy

**Figure 9:**
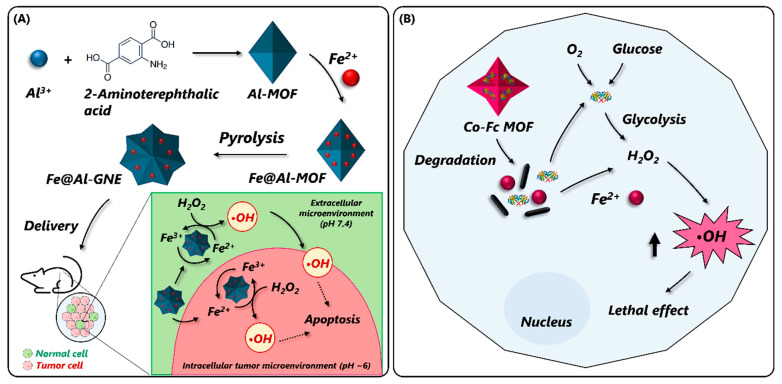
Chemodynamic activity of MOF-NZ. (**A**) Preparation and chemodynamic performance of Fe, Al, and N co-incorporated graphitic nanozyme (Fe/Al-GNE). Adapted with permission from [215] and (**B**) cascade enzymatic/Fenton reaction for elevated ^•^OH production in tumor cells. Adapted with permission from [217].

Elevated levels of reactive oxygen species (ROS) have been reported to control several aspects of tumor development and progression. However, considerably high concentrations of ROS can mediate cellular apoptosis. Therefore, tumor cells maintain a precise balance of their intracellular concentration by expressing high levels of antioxidants [212]. In chemodynamic therapy (CDT), a chemodynamic agent is used to selectively kill tumor cells by excess endogenous ROS generation. MOFs hold significant potential for CDT, wherein they serve as carriers for a wide range of therapeutic substances that are released to facilitate the treatment process. MOFs employ the Fenton/Fenton-like reaction, converting local H_2_O_2_ into cytotoxic ^•^OH radicals, thereby selectively eliminating tumor cells by utilizing endogenous ROS [213]. Within the acidic tumor microenvironment, the transition metal ions present in MOFs act as catalysts, enabling effective cancer therapy. The unique properties of MOFs, including stimuli-responsiveness and porosity, make them excellent candidates for CDT (Table 2). When selecting a chemodynamic agent, several factors should be considered. Firstly, it is essential to choose a specific tumor cell biomarker to minimize non-targeted killing and associated side effects. Additionally, pH sensitivity is crucial as the Fenton reaction occurs under acidic conditions [18,214]. Li et al. (2020) utilized the catalytic efficiency of iron-based Fenton agents by encapsulating Fe^2+^ in an aluminum (Al)-based MOF called MIL-101-NH_2_ to construct a Fe@Al-MOF graphitic nanozyme [215] (Figure 9A). The Al component acts as an electron pump, converting Fe^2+^ to Fe^3+^, while the graphitic sheet accelerates electron delivery, boosting the Fe^2+^/Fe^3+^ cycle regardless of pH. Consequently, the Fe@Al-MOF graphitic nanozyme rapidly and efficiently generates ^•^OH in both extracellular and intracellular microenvironments, inducing apoptosis in human liver tumor cells (SMMC-7721).

Tumor cells have a higher glucose consumption rate compared to normal cells due to their need for continuous cell proliferation and migration [216]. Fang et al. (2020) developed a Co-ferrocene MOF (Co-Fc MOF) with strong Fenton activity to utilize excess glucose in a murine tumor model [217]. They combined GOx with a Co-Fc MOF to create a cascade nanozyme (Co-Fc@GOx) for enhanced tumor treatment in murine breast cancer (4T1) cells. The high GOx loading capacity of MOF, along with its destabilization in the acidic tumor microenvironment, made it an ideal candidate to exert chemodynamic effects through the Fenton activity (Figure 9B). The increased level of H_2_O_2_ promotes the Fenton reaction, leading to the production of cytotoxic ^•^OH radicals. In another study, Li et al. (2020) developed a POX-mimic ferrimagnetic Zn-based MOF (Fe@ZIF-8) by employing a mineralization process [218]. Additionally, they embedded GOx into Fe@ZIF-8 to convert excess glucose in tumors into H_2_O_2_, leading to the production of highly toxic ^•^OH through a cascade reaction. Utilizing a magnetic field allowed for the precise delivery of MOF-NZs to specified locations, minimizing damage to normal cells. Aside from glucose, lactic acid (LA) plays a vital role in tumor microenvironments as a metabolic driver [219]. Researchers have developed a new treatment approach for selective tumor chemotherapy using an MOF-NZs system that is catalytic and pH-dependent, inspired by the high levels of LA found within tumors [220]. They achieved this by constructing a lactate oxidase (LOx)-immobilized Ce-benzenetricarboxylic acid (Ce-BTC) MOF, which leveraged the acidity and overexpression of LA in the tumor. This framework enabled the generation of highly toxic ^•^OH through a cascade reaction. Initially, LOx releases H_2_O_2_ from LA catalytically. Then, Ce-BTC, exhibiting POX-like activity, converts H_2_O_2_ into ^•^OH. Consequently, the ^•^OH effectively induces tumor apoptosis and cell death. In addition, the catalytic MOF selectively kills tumor cells while showing minimal side effects on normal organs.

The MOF-NZs strategy offers a novel approach to selective tumor chemotherapy, harnessing the distinct features of the tumor microenvironment such as acidic pH and abundant tumor biomarkers. By targeting this microenvironment and utilizing the cascade enzymatic reaction of MOF-derived cytotoxic free radicals, the CDT approach holds the potential to improve the efficacy of tumor-specific chemotherapy. However, as this field continues to develop, there are noteworthy aspects that may have been overlooked or merit further exploration. One essential consideration is the optimization of the chemodynamic reaction efficiency of MOF-NZs. Moreover, exploring the immunogenicity and potential immune responses triggered by MOF-NZs is vital for ensuring their safety and minimizing any adverse effects during chemodynamic therapy. Comprehensive studies on the interactions of these nanozymes with the immune system will contribute to their successful translation into clinical practices.

#### 4.2.2. Photodynamic Therapy

**Figure 10:**
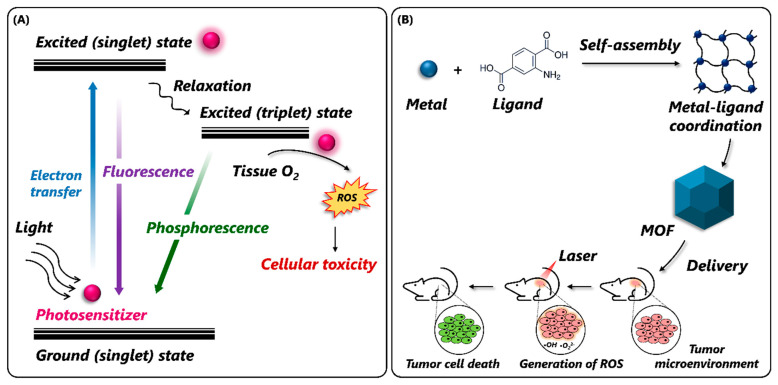
Photodynamic activity of MOF-NZs. (**A**) Jablonski diagram to represent the underlying photochemistry of photodynamic therapy in causing cellular toxicity. Adapted with permission from [223], and (**B**) construction of MOF-based PS and activation via laser irradiation for tumor treatment. Adapted with permission from [232].

Photodynamic therapy (PDT) involves combining molecular oxygen with a light-sensitive molecule called a photosensitizer (PS), resulting in an interaction between the PS and light [221]. A PS is applied to the tumor tissue, followed by exposure to a specific light wavelength. This process generates ROS such as singlet oxygen, superoxide radicals, ^•^OH radicals, and H_2_O_2_ [222]. These cytotoxic photoproducts cause damage and cell death in the target tissue. Previous generations of PSs had limitations, such as skin photosensitivity and intracellular self-aggregation. As illustrated in the well-known Jablonski diagram in Figure 10A, a cascade of biochemical events is triggered when these cytotoxic photoproducts arrive at the target tissue, causing damage and death to the cells. The first-generation PSs exhibited long-term skin photosensitivity, while the second generation required drug-delivery vehicles to enhance their efficacy. The third generation encountered challenges regarding intracellular self-aggregation. Meanwhile, a novel development in PDT is the use of MOFs as fourth-generation PSs [223].

MOFs have been extensively studied as drug delivery systems due to their porous structure that can accommodate a wide range of molecules including PSs. The use of MOFs as PSs in PDT represents a significant advancement and addresses some of the limitations faced by previous generations (Table 2). A chronological report on PS-based MOF development for photodynamic therapy was reported by Alves et al. (2021) [224]. Lu et al. (2014) reported a superior photoactivity of the PS porphyrin to treat head and neck cancer when it is part of the defined network of the MOF by avoiding molecule aggregation and self-quenching [225]. Since then, the phototoxicity effect of the MOF has been improved by changing the photosensitizer and inorganic metal unit [226,227,228].

Tumor hypoxia, caused by an inadequate and heterogeneous vascular network, is associated with poor clinical outcomes [229]. A study demonstrated that immobilizing Pt NPs on the PCN-224 MOF resulted in high stability and CAT-like activity in the PCN-224-Pt NP [230]. This compound overcomes the challenge of tumor hypoxia by generating singlet oxygen from H_2_O_2_. Another group developed MnFe_2_O_4_@MOF-NZ where the porphyrin-based MOF acted as a photosensitizer under laser irradiation while MnFe_2_O_4_ exhibited both CAT-like and glutathione POX-like activities [231]. This promoted singlet oxygen generation via the Fenton reaction in a concentration and time-dependent manner. Additionally, Liu et al. (2020) prepared Au NP-doped Fe-MOFs that protected and enhanced the GOx-like performance of the Au NPs [232]. When exposed to infrared light for 15 min, the Fe-based MOFs catalyzed the conversion of H_2_O_2_ to oxygen and subsequently ^•^OH, causing tumor cell death due to hypoxia (Figure 10B). The surface modification of MOFs using polymers like polyethylene glycol (PEG) provides excellent stability in physiological conditions. This modification holds great promise for MOF-NZs as a biodegradable and multifunctional therapeutic platform specifically targeting tumors for cancer treatment [233,234,235,236].

The combination of PDT and immunotherapy has recently emerged as a promising approach for treating metastatic cancer. PS-based MOFs have the potential to integrate PDT and immunotherapy into a single system by loading immunostimulatory agents that regulate immune checkpoints and modulate protein expressions [237,238]. This combination can significantly enhance the systemic tumor-specific immune response and offer clinical benefits for challenging-to-treat cancers. Studies have shown that MOF-PEG, with high PS loading, effectively produces singlet oxygen under light exposure for PDT. However, excessive photosensitizer amounts can lead to side effects. Therefore, the development of a PS that reduces glutathione levels while improving ROS concentration is crucial to enhance PDT efficacy against tumors. Zhang et al. reported the use of a Cu-metalated MOF (MOF-2) based on Cu^2+^ as the active center for PDT. Absorbing MOF-2 by breast cancer cells significantly reduced intracellular GSH levels, synergistically increased ROS concentration, and accelerated apoptosis.

The MOF-NZs have gained attention in cancer therapy in recent times due to their high cellular uptake, enhanced ROS generation, minimal side effects, and highly effective PDT [230,239]. Several challenges affecting clinical development, such as poor stability, low repeatability, water dispersion issues, and photosensitivity after treatment, have remained and are often overlooked. Although preliminary studies may show negligible toxicity of PS-based MOFs in vitro and in vivo, further understanding of their toxicity is necessary. Furthermore, in vivo studies should examine MOF distribution, metabolism, and excretion to assess its potential toxic metal accumulation. These analyses will guide therapeutic dosages during in vivo experiments and facilitate clinical research, establishing a benchmark for MOF-NZs.

#### 4.2.3. Photothermal Therapy

**Figure 11:**
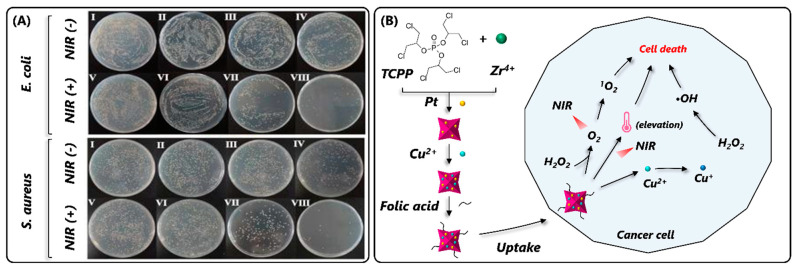
Photothermal activity of MOF-NZ. (**A**) Photographs of bacterial colonies with and without NIR irradiation. Adapted with permission from [242] and (**B**) layer-by-layer synthesis of MOF and photothermal effect in cancer cells. Adapted with permission from [246].

Photothermal therapy (PTT) is a medical treatment that utilizes light-absorbing materials, often nanoparticles, to convert absorbed light into heat. This localized increase in temperature is employed to selectively target and destroy diseased cells while minimizing damage to surrounding healthy tissues. Some common photothermal agents include carbon nanomaterials, metal nanoparticles, and organic dyes [240]. However, their application is limited due to the low photothermal conversion efficiency and difficult metabolism. Recently, there has been considerable interest in NZ-based PTT due to its potential to lower adverse effects and improve therapeutic efficiency (Table 2). MOF-NZs have drawn attention to their ability to mitigate bacterial infections via PTT [241]. In a recent study, an MOF-NZ integrating ZIF-8 with Pt NPs was developed for antibacterial activity against *E. coli* and *S. aureus* [242]. The developed MOF-NZ composite also incorporated PEG to prevent aggregation and ensure excellent stability. The Pt NP coating rendered MOFs with POX-like catalytic activity in the resulting PEG@Zn/Pt–CN which greatly improved photothermal conversion at ambient temperature. The Zn^2+^ ions released from ZIF-8 and the POX-like activity of PEG@Zn/Pt–CN disrupted the bacterial membrane and caused protein denaturation, respectively. The photothermal characteristic was confirmed by employing an 808 nm near-infrared laser (NIR) that transformed light energy into thermal energy for effective eradication (~98–99%) of *E. coli* and *S. aureus* (Figure 11A). In another study, a photothermal hydrogel composed of Zr-ferrocene (Zr-Fc) MOF was utilized for antibacterial therapy [243]. The integration of 2D Zr-Fc MOF-NZ with hydrogel enabled better control over the photothermal properties and thereby enabled precise antimicrobial therapy. Since the MOF-NZ hydrogel has superior POX-like activity, it could effectively eliminate *E. coli* and *S. aureus* bacteria by generating toxic ^•^OH radicals under 808 nm NIR light irradiation. The photothermal property of the MOF enabled the acceleration of cellular temperature and induced irreversible bacterial death.

Besides antibacterial therapy, PTT is an important technique to minimize cancer [244,245]. In a study, a Cu^2+^-doped MOF-NZ was developed using a Pt and folic acid (FA) coating via a layer-by-layer approach [246]. The attached FA on the surface selectively targets breast cancer cells, while Pt induces in situ generation of ROS, and Cu^2+^ produces a photothermal effect under 650 nm light exposure (Figure 11B). Ultimately, the catalytic capabilities of the MOF-NZ system effectively eliminate cancer cells with minimal side effects, demonstrated both in vitro and in vivo. In another study, amine-functionalized MOFs (NH_2_-MOFs) were used as a template to fabricate chlorin e6 (Ce6) in porous gold nanoshells (NH_2_-MOFs@Au) to kill cancer cells [247]. Additionally, the heat conversion capability of black phosphorus quantum dots was utilized to make a hybrid MOF against hypoxic tumor cells [248]. This hybrid MOF performed a catalase-like activity to sequentially convert H_2_O_2_ to O_2_ and O_2_ to ^1^O_2_.

The integration of MOF-NZs in the realm of PTT has emerged as a promising avenue with significant potential for biomedical applications. The unique properties of MOF-NZs, including their ability to mimic enzymatic activities and generate heat under specific conditions, make them valuable contributors to the field of PTT. However, amid the optimism surrounding MOF-NZs, certain aspects warrant careful consideration and further investigation. One notable aspect is the optimization of the photothermal conversion efficiency of MOF-NZs. Ensuring maximal heat generation while minimizing potential side effects or unintended damage to surrounding tissues is crucial for their successful translation into clinical applications. The potential long-term stability of MOF-NZs during repeated photothermal cycles also deserves attention. Understanding their durability and performance over extended periods is essential for establishing their reliability and effectiveness in sustained therapeutic applications. Furthermore, the environmental impact of MOF-NZ synthesis and disposal should not be overlooked.

#### 4.2.4. Anti-Inflammatory Role of MOF-NZ

**Figure 12:**
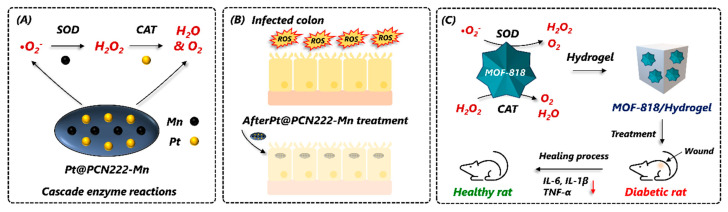
Anti-inflammatory activity of MOF-NZs. (**A**) Dual enzymatic performance of PCN222-Mn MOF-NZ. (**B**) The ROS scavenging capacity of PCN222-Mn MOF via cascade reactions. Adapted with permission from [252]; and (**C**) illustration of MOF-818/hydrogel preparation having SOD and CAT activity and wound healing in diabetic rats via downregulation of pro-inflammatory cytokines. Adapted with permission from [255].

Cells produce ROS as a necessary intermediate during cellular metabolism. A healthy cell balances the cytoplasmic ROS via various antioxidant mechanisms as excess ROS production may interfere with the cellular antioxidant system, damage biomolecules, and cause inflammation [249,250,251]. To mitigate these issues, MOF-NZs have gathered newfound interest due to their potential to modulate inflammatory responses and mitigate excessive inflammation. MOF-NZs can act as catalysts in various reactions, including those involved in regulating inflammatory pathways. By mimicking the functions of natural enzymes, they can influence the production and activity of key signaling molecules involved in inflammation [252,253,254,255,256,257]. In one such study, an integrated cascade nanozyme was developed by embedding Pt NPs in a PCN222-Mn MOF to eliminate ROS and effectively treat inflammatory bowel disease in mice [252]. The developed MOF-NZ could mimic the functions of SOD and CAT activities efficiently and demonstrate synergistic ROS scavenging ability for inflammatory treatment in both in vitro and in vivo experiments (Figure 12A,B). In another recent study, a dual enzyme-mimicking bimetallic Cu-TCPP MOF was fabricated by incorporating Mn (II) [253]. The developed Cu-TCPP-Mn MOF was ~100 nm in size and could mimic the SOD and CAT activities to produce H_2_O_2_ via cascade reactions. The MOF-NZ showed excellent target-specific ROS scavenging activity followed by demonstrating anti-inflammatory effects in myocardial injury. Similarly, the SOD-like enzyme activity of nickel in a Ni-based MOF was employed in a study to scavenge various free radicals via an anti-inflammatory reaction [254]. The Ni-MOF significantly inhibited the expression of pro-inflammatory cytokine (interleukin 6) and promoted anti-inflammatory cytokine (interleukin 10). It is important to understand the long-term stability and biocompatibility of MOF-NZs in complex biological environments. Hence, understanding the MOF-NZ’s behavior over extended periods and their potential interactions with the immune system is vital for predicting their efficacy and safety in practical anti-inflammatory applications. To mitigate chronic infection in diabetic wounds, an anti-oxidative MOF-818 NZ was developed to modulate oxidative stress [255]. The prepared MOF had excellent stability in water and displayed intrinsic SOD- and CAT-like activities that could scavenge the ROS of the infected wound (Figure 12C). Besides pure materials, hydrogel-based therapy has gained attraction in recent times due to biocompatibility and controlled release of therapeutic agents. Given this, Chen et al. (2023) developed a 3D-printed MOF nanozyme hydrogel having dual enzymatic activities for diabetic wound healing [256]. In the type 1 diabetic rat model, 1 cm^2^ size diabetic wounds were fully healed within 21 days after the intraperitoneal delivery of the MOF (60 mg/kg).

The application of MOF-NZs in the context of anti-inflammatory therapy presents a compelling avenue for biomedical research. These nanozymes, leveraging their unique ability to mimic enzymatic activities, offer a potential solution for mitigating inflammatory responses (Table 2). Despite the promising potential of MOF-NZs in anti-inflammatory therapy, certain challenges and considerations need careful attention. These include ensuring the specificity of the nanozymes for inflammatory targets, understanding their long-term stability in complex biological environments, and evaluating their potential immunomodulatory effects on the overall immune system. Therefore, comprehensive studies on the impact of MOF-NZs on inflammatory mediators and immune cell function will contribute to a more nuanced understanding of their anti-inflammatory mechanisms.

#### 4.2.5. Antimicrobial Activity

**Figure 13:**
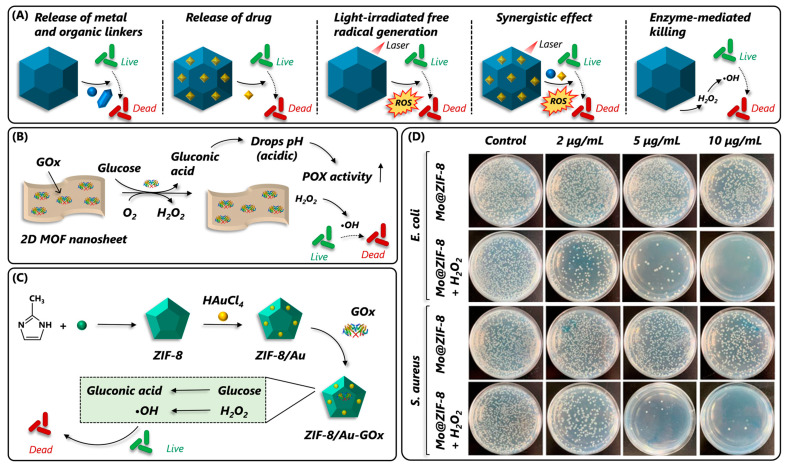
Antimicrobial activity of MOF-NZs. (**A**) Key roles of MOF-mediated antibacterial mechanism. (**B**) Design and antibacterial mechanism of 2D MOF/GOx as self-activated cascade reagent. Adapted with permission from [267]; (**C**) construction and reaction of ZIF8/Au-Gox for antibacterial treatment. Adapted with permission from [265]; (**D**) bacterial viability of antibacterial nanozyme (Mo@ZIF-8) with a low concentration of H_2_O_2_ (10^−5^ M). Reproduced with permission from [269].

MOF-NZs emerge as a promising tool in antibacterial therapy, showcasing notable enzyme-like activity and a consistent release of metal ions. These materials mimic natural enzymes such as peroxidase or oxidase, generating powerful free radicals with significant antibacterial effects. Leveraging the catalytic properties of MOF-NZs enables precise targeting and damage to bacterial cells. The resulting ROS induces oxidative stress, harming the bacterial cell membrane, DNA, and proteins, ultimately causing bacterial cell death [23]. This ROS-mediated antibacterial mechanism proves effective against a broad spectrum of bacteria. While natural enzymes catalyze ROS production, their practical application is hindered by high costs, complex purification processes, poor stability, and challenging recycling. Hence, the quest for enzyme-like materials capable of replicating these natural enzyme activities remains imperative. In recent years, MOFs have garnered significant attention for their potential in antibacterial therapy, addressing bacterial attachment prevention, growth inhibition, and direct bacterial eradication [258,259,260,261,262]. Figure 13A outlines the key roles of MOFs in bacterial killing, including the following: (a) releasing metal ions and organic linkers for bacterial elimination, (b) acting as carriers for antimicrobial and small molecule drugs, (c) utilizing photosensitive MOFs to induce bacterial death through heat and free radical production under light exposure, (d) employing a synergistic approach with multiple active compounds for bacterial killing, and (e) mimicking enzymes to combat bacteria. This review specifically emphasizes the recent advancements of MOF-NZs in antibacterial therapy.

Certain MOFs, including those based on Cu, Zn, and metalloporphyrins, demonstrate enzyme-like properties, making them potent antibacterial agents [263]. Essentially, these MOFs induce the intracellular release of reactive oxygen species (ROS), leading to bactericidal effects through oxidative stress [29,264,265]. MOFs with simulated OX and POX activities are commonly used to generate a substantial amount of ROS, causing intense oxidative stress for efficient bacterial disinfection [26]. Nanomaterials, particularly a hybrid 2D MOF with ultra-small Au nanoparticles, show remarkable POX-like activity, decomposing H_2_O_2_ into toxic ^•^OH and exhibiting excellent antibacterial properties against both Gram-negative (*E. coli*) and Gram-positive (*Staphylococcus aureus*) bacteria [266]. In order to achieve a synergistic effect on bacteria, Liu et al. designed a nano catalyst by combining 2D MOF Cu-TCPP(Fe) with GOx and demonstrated high antibacterial efficacy against *E. coli* and *S. aureus* through the conversion of glucose into abundant H_2_O_2_ and gluconic acid (Figure 13B) [267]. In another study, GOx was integrated with NH_2_-MIL-88B(Fe) to combat methicillin-resistant *S. aureus* (MRSA) via self-activated cascade reactions [268]. Apart from these, ZIF-based MOF-NZs have also been investigated for antibacterial performance. In one such example, Wang et al. developed an MOF-NZ by integrating ZIF-8 with GOx and Au NPs and using it for highly efficient bacterial eradication through a cascade catalytic reaction [265]. This ZIF8/Au-GOx demonstrated excellent POX-like activity, which consequently allowed it to exhibit tremendous antibacterial activity at low concentrations (e.g., 8 μg/mL for *E. coli* and 4 μg/mL for *S. aureus*) by releasing ROS and Zn^2+^ (Figure 13C). Another ZIF-based MOF-NZ was developed by doping molybdenum (Mo) in ZIF-8 MOF (Mo@ZIF-8) [269]. The fabricated MOF-NZ mimicked POX activity, which triggered ^•^OH radical production to exert antibacterial activity (~99%) against both Gram-negative (*E. coli*) and Gram-positive (*S. aureus*) bacteria (Figure 13D). Lysozyme, an FDA-approved food preservative, can hydrolyze peptidoglycan bonds in bacterial cell walls; however, free lysozyme has limited antibacterial efficacy due to instability. Reportedly, lysozyme immobilization on a solid matrix could enhance its stability and hence improve antibacterial activity. Prompted by this, Nong et al. developed a novel antibacterial agent by immobilizing lysozyme in MIL-88B(Fe) MOFs [270]. Consequently, under near-infrared irradiation, the lysozyme layer degraded peptidoglycan of the bacterial cell wall and disrupted bacterial cell membranes via a glutamine transaminase enzymatic reaction.

The urgent need for effective antimicrobial agents to control pathogenic microbes can potentially be addressed by MOF-NZ, which plays a crucial role in antibacterial therapy by mimicking enzymatic activity and generating ROS for bacterial cell death (Table 2). Combining antibacterial metals with enzymes in therapy enhances effectiveness and provides a strategic solution for improving bacterial eradication [265]. However, one aspect deserving attention is the specificity of MOF-NZs in targeting bacterial cells while minimizing its impact on healthy cells. Fine-tuning the selectivity of these nanozymes will enhance their effectiveness in eradicating bacterial infections while preserving the integrity of surrounding tissues. The long-term stability and biocompatibility of MOF-NZs in complex biological environments should be thoroughly investigated. Moreover, the potential for MOF-NZs to induce bacterial resistance over time should be carefully examined. Continuous exposure to these nanozymes may lead to adaptive responses in bacteria, necessitating comprehensive studies to assess the risk of resistance development and strategies to mitigate such challenges.

**Table 2:** Therapeutic applications of MOF-based nanozymes.

MOF	Enzyme-Mimic Activity	Applications	Notable Features	Reference
Co–Fc NMOF	GOx	CDT	▪ Effective delivery of Fenton agent (ferrocene) and cargo (GOx) ▪ Demonstrates in vitro and in vivo anticancer effect ▪ Degradable characteristic in an acidic environment ▪ No in vitro toxicity to normal cells ▪ Dose-dependent cytotoxicity to 4T1 cancer cells ▪ In vivo anticancer effect (4T1 tumor-bearing mice)	[217]
Au/FeMOF	GOx	CDT	▪ Au NP fabrication improves the stability of MOF ▪ Intracellular glucose can be oxidized by Au NPs ▪ Systemic toxicity to tumor ▪ IC_50_ value 0.31 ± 0.04 µg mL^−1^ ▪ Half-life 4.27 ± 0.36 h ▪ In vivo antitumor effect (HepG2 tumor-bearing mice)	[271]
UCNP–iron porphyrinic MOF NPs@Au	CAT	CDT	▪ AuNP decoration stabilizes MOF and serves as radiosensitizers ▪ Suppresses the tumor growth ▪ Negligible systemic toxicity ▪ Biodegradable ▪ PEGylation to prevent aggregation ▪ Anticancer efficacy against U-87 MG cells ▪ 15% inhibition of cell proliferation ▪ Biodistribution in U-87 MG tumor-bearing female nude mice	[272]
MIL-88A	CAT	PDT	▪ Catalyze H_2_O_2_ into O_2_ within 10 min ▪ Generate O_2_ in a hypoxic situation (660 nm laser) ▪ >90% cell viability in s U-87 MG cells	[239]
PCN@Pt@PCN−Au−FA	CAT and GOx	PDT	▪ O_2_-dependent PDT (671 nm laser) ▪ Prevention of tumor recurrence and metastasis ▪ Pt NPs effectively convert intracellular H_2_O_2_ ▪ In vivo anticancer effect (4T1 tumor-bearing mice) ▪ Efficient accumulation in tumor ▪ Excellent biocompatibility and intratumoral O_2_-evolving ability ▪ No noticeable side effects	[273]
DBBC–UiO	SOD	PDT	▪ Enable massive O_2_^−^ under NIR irradiation (750 nm laser) ▪ Recycled O_2_ enhances O_2_^−•^ generation ▪ Antitumor performance ▪ Exhibits a good photoacoustic response ▪ 94% MCF-7 cell death with laser irradiation ▪ High tumor accumulation (≈13% ID/g in 24 h) ▪ In vivo antitumor performance (MCF-7 mice) ▪ High biocompatibility	[274]
Fe-MOF	GOx	PDT	▪ AuNP coating protects the GOx activity ▪ NIR light-enhanced GOx-like activity (in vitro and in vivo) ▪ Increase endogenous H_2_O_2_ and ROS generation (A549 cell) ▪ Generate local heat upon NIR light irradiation ▪ Good stability in cell culture media ▪ No obvious pathological change	[232]
Au NCs@PCN	SOD	PTT	▪ Excellent ROS generation and photothermal effects ▪ Effective killing effect on MRSA (95%) and Amp^r^ *E. coli* (91%) ▪ Reduced wound coverage by 2.7% in diabetic rats (in 21 days) ▪ 808 nm laser irradiation ▪ PI3K/AKT and CREB pathways accelerate wound healing ▪ No obvious cytotoxicity to HUVECs and HACAT cells	[275]
Cu-TCPP-Mn	SOD and CAT	Anti-inflammatory	▪ Bimetallic MOF mimics the cascade enzymatic activities ▪ Synergistic ROS scavenging and anti-inflammation effect ▪ Effective for myocardial injury treatment	[253]
Ni-MOF	SOD	Anti-inflammatory	▪ Slow release of Ni reduced toxicity in cell ▪ Improved wound healing in rat ▪ An effective choice for injectable and sprayable medical dressings ▪ Long-term therapeutic effects ▪ Promote fibroblast migration and angiogenesis	[254]
MOF-818	SOD and CAT	Anti-inflammatory	▪ MOF was integrated with a hydrogel ▪ Effective antioxidative system for chronic wound healing ▪ Scavenge ROS continuously to moderate the oxidative stress in diabetic rats’ wound ▪ Stable for more than 6 months ▪ Biocompatible ▪ Sustainable treatment	[255]
ZIF-8	GOx	Antibacterial	▪ Integration of GOx and Au NPs support cascade catalytic reaction ▪ Effectively catalyze the glucose to produce H_2_O_2_ ▪ Effective bactericidal performance via ROS ▪ Eradicate bacteria at low concentrations (8 μg/mL for *E. coli* and 4 μg/mL for *S. aureus*) ▪ Promote wound healing process in *S. aureus*-infected mice	[265]
MIL-88B	POX	Antibacterial	▪ Delivery of photothermal agent ▪ Catalytic activity regulated by NIR laser irradiation (808 nm) ▪ Biofilm penetration capacity ▪ MRSA bacterial disinfection via catalytic reactions ▪ MIC value in MRSA was 50 μg/mL ▪ Negligible hemolytic activity and cytotoxicity ▪ In vivo disinfection in MRSA-infected wound model	[276]
Cu-TCPP(Fe))	POX and GOx	Antibacterial	▪ Self-activated nanocatalyst ▪ Antimicrobial activity against *E. coli* and *S. aureus* ▪ 88% and 90% bacteria inactivation rate for *E. coli* and *S. aureus*, respectively ▪ Promote wound healing (56.5% reduction) ▪ Stable in media (7 days)	[267]
IRMOF-3	POX	Antibacterial	▪ Antibacterial effects against *E. coli* and *S. aureus* ▪ Produce ^•^OH in low H_2_O_2_ dose ▪ Exhibits stable catalytic activities within pH 3-6.5 ▪ High catalytic activities between 20 and 50 °C ▪ Display catalytic activities within 30 min reaction time ▪ Exhibit excellent biocompatibility and hemocompatibility ▪ Maintain >90% cell viability in HUVECs and L-02 cells ▪ 76% and 72% antibacterial rates for *E. coli* and *S. aureus*, respectively ▪ Strong anti-biofilm effects (83% and 91% biofilm inhibition rate of *E. coli* and *S. aureus*, respectively) ▪ Promote wound healing (KM mice)	[277]
ZIF-8	POX	Antibacterial	▪ Effective antibacterial capability against both Gram-negative and Gram-positive bacteria ▪ 99.2% and 99.4% inhibition efficiency in *E. coli* and *S. aureus*, respectively ▪ Excellent antibacterial capability with minimal H_2_O_2_.	[269]
UiO-66	POX	PDT, PTT, antibacterial	▪ Nanozyme activity generates ROS (808 nm light) ▪ Ampicillin resistant *E. coli* and MRSA ▪ Negligible toxicity ▪ Promote wound healing	[278]

## 5. Challenges and Prospects

MOF-NZs are emerging with promising applications in disease monitoring, food safety and quality testing, and therapeutic domains. The present review sheds light on examples of MOFs and their composites that could mimic natural enzymatic activities and discusses various factors that regulate the MOF-NZ’s stability and catalytic performance. It further gives a deep insight into the utilization of MOF-NZs for biosensing and therapeutic applications. While MOF-NZs exhibit tremendous potential, several challenges remain that need to be addressed in order to exploit their full potential. To develop high-performing MOF-NZs for their potential applications in clinical diagnosis and treatment, the following challenges should be considered.

The design and development of MOF-NZs with targeted catalytic activity pose a significant challenge. Most existing MOF-NZs suffer from notably low catalytic activity due to their relatively large size and limited accessible active sites. Despite extensive efforts towards enhancing the intrinsic nanozyme activity of MOF, the improvements achieved so far remain unsatisfactory. Consequently, their catalytic capacity lags significantly behind that of natural enzymes. Addressing this issue necessitates more efficient approaches to significantly enhance the catalytic activity of MOF-NZs. Recently, the introduction of single-atom metal species into the MOF and MOF derivatives has been shown to substantially improve the nanozyme activity [279]. However, achieving the single-atomization of active sites in MOF nanozymes while preserving their desirable structures and properties poses a significant challenge. In parallel, the integration of MOF-NZs with optofluidics may present another avenue to enhance the optical signal readout and provide real-time monitoring of dynamic biological systems, essential for health monitoring and biomedical tests [280,281].

The specificity of MOF-NZs is another significant challenge that requires careful consideration across diverse applications. Since the intrinsic lack of specificity may result in undesirable side reactions and reduced efficiency in targeted catalysis, conjugating nanozymes with biological molecules like aptamers [185] and antibodies [159,282] can be a crucial approach in addressing this limitation. These biological MREs can not only confer target-specific binding properties to MOF-NZs but also enhance their selectivity for specific substrates. Despite being useful, incorporating biological MREs can substantially increase the cost of MOF-NZs and compromise their stability under harsh conditions. In view of this, developing molecularly imprinted polymers, also referred to as “plastic antibodies”, may be an ideal alternative for enhancing MOF-NZ sensitivity by creating substrate-binding cavities [283,284].

MOF-NZs have garnered global attention for their potential in biosensing and therapeutic applications. However, as discussed above, most studies have only exploited MOFs with POX- and OX-like activities. In order to truly meet the natural enzymes’ potential for widespread applications, extensive efforts are required to explore MOFs with other catalytic activities. Another aspect that requires significant attention is the development of stimuli-responsive MOF-NZs. While continued efforts are being made in this direction, [285] the research in this field is still very limited and requires extensive efforts in future endeavors.

To date, the advancement of nanozymes primarily relies on a trial-and-error strategy, demanding significant time and resources. With the tremendous advancement in computer science, the application of computer-aided high-throughput screening has emerged as an efficient strategy for evaluating performance and rapidly identifying desired materials. The integration of artificial intelligence and sophisticated computational techniques, encompassing machine learning and simulations, holds the potential to significantly enhance the efficient design of MOF-NZs with specific functionalities. Additionally, the concept of directed evolution in enzymes can be extended to MOF-NZs, allowing for the deliberate development of desired traits by emulating the evolutionary process.

Due to its inherent toxicity, the inadequate understanding of the MOF precursor transition to its derivatives, owing to its inherent complexity, represents another aspect. A thorough understanding of the high-temperature pyrolysis procedure could offer advantages in attaining meticulous control over porosity, active sites, coordination environments, and localized configurations crucial for synergistic and cascade reactions.

Enzyme immobilization in MOFs offers promising avenues for catalysis; however, it comes with several inherent limitations. One major challenge lies in the potential decrease in catalytic activity due to hindered mass transfer, a consequence of the immobilization process. The stability of MOFs is often compromised by the reversibility of coordination bonds, impacting the durability of the immobilized enzymes. Additionally, the difficult functionalization and relatively low stability of natural enzymes further hinder the overall catalytic performance of enzyme@MOF-based systems. Given this, achieving a delicate balance between effective immobilization, catalytic activity preservation, and sustained stability is imperative in overcoming these limitations and unlocking the full potential of enzyme@MOF-based systems.

Despite the MOF-NZ field being in a nascent stage, the accumulation of reports on biomedical applications has been overwhelming due to their easy preparation, tandem catalysis, excellent thermal stability, and high enzyme loading efficiency. Furthermore, their modular physicochemical properties enable the ease of loading of photosensitizers and chemotherapeutic drugs for numerous therapeutic applications. While the success of MOF-NZs is commendable for its short span, future studies should focus on the rational design of MOF-NZs, exploring the underlying mechanism behind catalysis, improving their stability in biological fluids, and understanding their pharmacokinetic behavior.

## Abbreviations

^•^OH	Hydroxyl radical	EC	Electrochemical	PDT	Photodynamic therapy
1D	One-dimensional	GOx	Glucose oxidase	PEG	Polyethylene glycol
2D	Two-dimensional	HRP	Horse-radish peroxidase	POC	Point-of-care
ABTS	2,2-azinobis(3-ethylbenzothiazoline)-6-sulfonic acid	LA	Lactic acid	POX	Peroxidase
AChE	Acetylcholinesterase	LOD	Limit of detection	PS	Photosensitizer
AuNPs	Gold nanoparticles	LOx	Lactate oxidase	PSA	Prostate cancer antigen
BA	Boronic acid	MIL	Material Institute of Lavoisier	PTT	Photothermal therapy
BChE	Butyrylcholinesterase	MNP	Metal nanoparticles	QDs	Quantum dots
CAT	Catalase	MOF-NZs	MOF-based nanozymes	ROS	Reactive oxygen species
CDT	Chemodynamic therapy	MOFs	Metal–organic frameworks	SAN	Single-atom nanozyme
Ce-BTC	Cerium-benzenetricarboxylic acid	MRSA	Methicillin-resistant *S. aureus*	SDM	Sulfadimethox
ChOx	Cholesterol oxidase	NIR	Near-infrared laser	SOD	Superoxide dismutase
CNTs	Carbon-nanotubes	NZs	Nanozymes	TCPP	Tetrakis(4-carboxyphenyl)porphyrin
DPV	Differential pulse voltammetry	OPD	O-phenylenediamine	TMB	3,3′,5,5′-tetramethylbenzidine
DSNC	Double-shelled hybrid nanocage	PCN	Porous coordination network	Zr-Fc	Zr-ferrocene
